# Impact of receiving recorded mental health recovery narratives on quality of life in people experiencing psychosis, people experiencing other mental health problems and for informal carers: Narrative Experiences Online (NEON) study protocol for three randomised controlled trials

**DOI:** 10.1186/s13063-020-04428-6

**Published:** 2020-07-20

**Authors:** Stefan Rennick-Egglestone, Rachel Elliott, Melanie Smuk, Clare Robinson, Sylvia Bailey, Roger Smith, Jeroen Keppens, Hannah Hussain, Kristian Pollock, Pim Cuijpers, Joy Llewellyn-Beardsley, Fiona Ng, Caroline Yeo, James Roe, Ada Hui, Lian van der Krieke, Rianna Walcott, Mike Slade

**Affiliations:** 1 School of Health Sciences, Institute of Mental Health, University of Nottingham Innovation Park, Triumph Road, Nottingham, NG7 2TU UK; 2 Division of Population Health, Health Services Research & Primary Care, University of Manchester, Oxford Road, Manchester, M13 9PL UK; 3 London School of Hygiene and Tropical Medicine, Keppel St, Bloomsbury, London, WC1E 7HT UK; 4 Centre for Primary Care & Public Health, Pragmatic Clinical Trials Unit, Queen Mary University of London, 58 Turner St, London, E1 2AB UK; 5 NEON Lived Experience Advisory Panel, Nottingham, UK; 6 Department of Informatics, King’s College London, Bush House, 30 Aldwych, London, WC2B 4BG UK; 7 School of Health Sciences, Queens Medical Centre, Nottingham, NG7 2UH UK; 8 Department of Clinical, Neuro and Developmental Psychology, Amsterdam Public Health Research Institute, Vrije Universiteit Amsterdam, Amsterdam, the Netherlands; 9 National Institute for Health Research, ARC East Midlands, University of Nottingham, Triumph Road, Nottingham, NG7 2TU UK; 10 University Medical Center Groningen, University Center of Psychiatry, University of Groningen, Hanzeplein 1, Groningen, 9713 GZ The Netherlands; 11 Department of Digital Humanities, King’s College London, Strand, London, WC2R 2LS UK

**Keywords:** Randomised controlled trial, Pragmatic trial, Recovery narratives, Recovery stories, Quality of life, MANSA, Psychosis, Carers, Mental health

## Abstract

**Background:**

Mental health recovery narratives have been defined as first-person lived experience accounts of recovery from mental health problems which refer to events or actions over a period of time and which include elements of adversity or struggle, and also self-defined strengths, successes or survival. They are readily available in invariant recorded form, including text, audio or video. Previous studies have provided evidence that receiving recorded recovery narratives can provide benefits to recipients.

This protocol describes three pragmatic trials that will be conducted by the Narrative Experiences Online (NEON) study using the NEON Intervention, a web application that delivers recorded recovery narratives to its users. The aim of the NEON Trial is to understand whether receiving online recorded recovery narratives through the NEON Intervention benefits people with experience of psychosis. The aim of the NEON-O and NEON-C trials is to evaluate the feasibility of conducting a definitive trial on the use of the NEON Intervention with people experiencing non-psychosis mental health problems and those who care for others experiencing mental health problems respectively.

**Methods:**

The NEON Trial will recruit 683 participants with experience of psychosis. The NEON-O Trial will recruit at least 100 participants with experience of non-psychosis mental health problems. The NEON-C Trial will recruit at least 100 participants with experience of caring for others who have experienced mental health problems. In all three trials, participants will be randomly allocated into one of two arms. Intervention arm participants will receive treatment as usual plus immediate access to the NEON Intervention for 1 year. Control arm participants will receive treatment as usual plus access to the NEON Intervention after 1 year. All participants will complete demographics and outcome measures at baseline, 1 week, 12 weeks and 52 weeks. For the NEON Trial, the primary outcome measure is the Manchester Short Assessment of Quality of Life at 52 weeks, and secondary outcome measures are the CORE-10, Herth Hope Index, Mental Health Confidence Scale and Meaning in Life Questionnaire. A cost-effectiveness analysis will be conducted using data collected through the EQ-5D-5 L and the Client Service Receipt Inventory.

**Discussion:**

NEON Trial analyses will establish both effectiveness and cost-effectiveness of the NEON Intervention for people with experience of psychosis, and hence inform future clinical recommendations for this population.

**Trial registration:**

All trials were prospectively registered with ISRCTN. NEON Trial: ISRCTN11152837. Registered on 13 August 2018. NEON-C Trial: ISRCTN76355273. Registered on 9 January 2020. NEON-O Trial: ISRCTN63197153. Registered on 9 January 2020.

## Trial information summary

**Table:** 

**Primary trial registrations**	All trials registered prospectively with ISRCN. NEON Trial: ISRCTN11152837, registered 13 August 2018, http://www.isrctn.com/ISRCTN11152837 NEON-C Trial: ISRCTN76355273, registered 9 January 2020, http://www.isrctn.com/ISRCTN76355273. NEON-O Trial: ISRCTN63197153, registered 9 January 2020, http://www.isrctn.com/ISRCTN63197153.
**Secondary identifying numbers**	IRAS ID: 249015 REC ref.: 19/EM/0326
**Source of monetary support**	National Institute for Health Research (NIHR) Programme Grant for Applied Research (RP-PG-0615-20016)
**Primary Sponsor**	Nottinghamshire Healthcare NHS Foundation Trust Contact: Mark Howells Duncan Macmillan House, Porchester Road, Nottingham NG3 6AA, 0115 9691300 Mark.Howells@nottshc.nhs.uk or Research@nottshc.nhs.uk
**Secondary Sponsor**	Not applicable
**Chief Investigator**	**Professor Mike Slade** Institute of Mental Health, University of Nottingham Innovation Park, Triumph Road, Nottingham, NG7 2TU m.slade@nottingham.ac.uk
**Contact for public enquiries**	**Stefan Rennick-Egglestone** Institute of Mental Health, University of Nottingham Innovation Park, Triumph Road, Nottingham, NG7 2TU stefan.egglestone@nottingham.ac.uk
**Contact for scientific enquiries**	**Professor Mike Slade** Institute of Mental Health, University of Nottingham Innovation Park, Triumph Road, Nottingham, NG7 2TU m.slade@nottingham.ac.uk
**Public title**	NEON (Narrative Experiences Online) study: trials of an online intervention
**Scientific title**	NEON (Narrative Experiences Online) study: trials of an online intervention
**Countries of recruitment**	England
**Health condition(s) or problem(s) studied**	NEON Trial: Psychosis NEON-O Trial: Non-psychosis mental health problems NEON-C Trial: Not a study of a health condition
**Interventions**	Intervention arm: treatment as usual plus access to online recovery narratives for 1 year Control arm: treatment as usual for 1 year, followed by access to recorded recovery narratives
**Inclusion criteria**	See main body of protocol
**Study type**	All trials are interventional, with no masking and 1:1 randomised allocation using a sequence generated through permuted blocks randomisation
**Date of first enrolment**	9th March 2020
**Target sample size**	NEON Trial: 683 NEON-C Trial: 100 NEON-O Trial: 100
**Recruitment status**	Recruitment opened same day as protocol submission
**Primary outcome**	Manchester Short Assessment of Quality of Life at 52 weeks after baseline.
**Secondary outcomes**	CORE-10, Herth Hope Index, Mental Health Confidence Scale, Meaning in Life Questionnaire, all at 52 weeks after baseline.
**Recruiting organisations**	**Nottinghamshire Healthcare NHS Foundation Trust** Principal investigator: Professor Mike Slade m.slade@nottingham.ac.uk Principal contact: Stefan Rennick-Egglestone stefan.egglestone@nottingham.ac.uk **Cornwall Partnership NHS Foundation Trust** Principal Investigator: Ruth Bishop ruth.bishop2@nhs.net Principal contact: Alan Beattie alan.beattie1@nhs.net **Derbyshire Healthcare NHS Foundation Trust** Principal Investigator: Dr. Soma Datta somadatta@nhs.net Principal contact: Andy Dingwall andy.dingwall@nhs.net **Devon Partnerships NHS Foundation Trust** Principal Investigator: Dr. Zara Bowling zara.bowling@nhs.net Principal contact: Christina Burke-Trees c.burke-trees@nhs.net **East London NHS Foundation Trust** Principal Investigator: Professor Stefan Priebe s.priebe@qmul.ac.uk Principal contact: Zivile Jakaite zivile.jakaite@nhs.net **Leicestershire Partnership NHS Trust** Principal Investigator: Dr. Fabida Noushad Fabida.Noushad@leicspart.nhs.uk Principal contact: Dave Clarke dave.clarke@leicspart.nhs.uk **Lincolnshire Partnership NHS Foundation Trust** Principal Investigator: Christine Coupar Christine.coupar@lpft.nhs.uk Principal contact: Tracy McCranor Tracy.McCranor@lpft.nhs.uk **North East London NHS Foundation Trust** Principal Investigator: Eilis Quinlan Eilis.Quinlan@nelft.nhs.uk Principal contact: Ana Cardoso Ana.Cardoso@nelft.nhs.uk **Oxford Health NHS Foundation Trust** Principal Investigator: Dr. Pamela Kaushal Pamela.Kaushal@oxfordhealth.nhs.uk Sub Principal Investigator and principal contact: Taneesha Jones-Seale Taneesha.JonesSeale@oxfordhealth.nhs.uk **Somerset Partnership NHS Foundation Trust** Principal Investigator and principal contact: Carinna Vickers Carinna.Vickers@sompar.nhs.uk **South London and Maudsley NHS Foundation Trust** Principal Investigator: Henrietta Webb-Wilson Henrietta.Webb-Wilson@slam.nhs.uk Principal contact: Carol Cooley carol.cooley@kcl.ac.uk **Sussex Partnership NHS Foundation Trust** Principal Investigator: Dr. Mark Hayward M.I.Hayward@sussex.ac.uk Principal contact: Kelly Wilson Kelly.Wilson@sussexpartnership.nhs.uk

## Background

Mental health *recovery narratives* have been defined as first-person lived experience accounts of recovery from mental health problems which refer to events or actions over a period of time and which include elements of adversity or struggle, and also self-defined strengths, successes or survival [1, 2]. They are referred to as recovery narratives in this protocol whilst recognising that this term is used elsewhere in healthcare research and practice, e.g. in narratives of recovery after a stroke [3]. Recovery narratives can be shared *live*, as part of social interactions with others, or they can be presented in *recorded* form, as invariant text, audio or video [4]. In this protocol, the person telling the story, in either form, is referred to as the *narrator*, and the person reading, watching, listening to or otherwise engaging with the story is referred to as the *recipient* [5].

Sharing of recovery narratives is common [6, 7]. Informal peer support, involving interactions between individuals with similar experiences of health problems, is one example of a naturally occurring relationship in which live recovery narratives can be narrated and received. Informal peer support can take place in person [8] or online [9]. In this century a new employment role of peer support worker or peer specialist has emerged in mental health systems internationally [10] which involves employing people in roles for which personal experience of mental health problems and recovery is a requirement. Intentional peer support has an empirical evidence base [11] and is being implemented globally [12]. A US national survey has identified helping others through the narrating of mental health recovery narratives as a feature of the work of peer specialists [13]. Peer support workers can create change through mechanisms such as role modelling of individual recovery [14]. Davidson et al. [15] have argued that the disclosure by a peer worker of their own transition to a “hero of their own self-journey” (p. 124) can instil hope in others. The growth of peer support work means that an increasing number of people living with mental health problems have access to live recovery narratives shared as part of a supportive relationship [15].

Access to recorded narratives is increasing [6, 7]. Substantial numbers of recorded recovery narratives are publicly available, distributed through mechanisms including books [16, 17], health service booklets [18], online collections [19] and digital media hosting services [20]. Creating narratives can also provide benefits for narrators [21], who might be motivated by sending messages of “hope, courage and survival” (p. 68) [22], a form of indirect emotional support [23]. Campaigns which aim to reduce stigma [24, 25], such as Bell Let’s Talk [26], have used recorded recovery narratives [27] as a mechanism for creating social contact between people with experience of mental health problems and others, drawing on long-standing evidence for social contact as an anti-stigma mechanism [28, 29]. Health material shared in anti-stigma campaigns can have a beneficial impact on help-seeking behaviour [30], a finding that is important when systematic review evidence shows that stigma can disrupt help-seeking behaviour [31]. Receiving a recovery narrative can provide personal inspiration [32], increase empathy and understanding [33], validate difficult personal experiences [34] or provide alternative forms of companionship at times of social isolation [35]. Receiving recovery narratives can also contribute to recipient distress, e.g. if the recipient feels angry or “out of place” through a perception that he/she has experienced greater hardship than a narrator [32].

The public availability of an increasing number of recorded recovery narratives is an opportunity to provide support to people through a new form of mental health intervention. Organisations such as Here to Help [36] and the Scottish Recovery Network [37] have already created online collections of recovery narratives with the explicit intent of supporting recovery in recipients. These might be seen as a specific initiative within a larger effort to incorporate digital healthcare technologies (DHTs) into mental health practice, motivated by known global challenges such as lengthy waiting lists for treatment [38], limited access to in-person mental health treatment in rural and remote communities [39–41] and the distress inherent in accessing in-person treatment for people experiencing social anxiety [42]. Systematic review evidence shows that DHTs can be effective at supporting self-management for long-term conditions [43], and because face-to-face contacts account for nearly 90% of healthcare interactions [44], then developing self-management skills might save health service resources as well as supporting better long-term outcomes [45].

A recent qualitative study using semi-structured interviews to investigate the impact of receiving live and recorded mental health recovery narratives for 77 participants identified three benefits specifically attributable to the supportive process of receiving recorded recovery narratives: obtaining access to narrators not available in everyday life; having control over when and how to access a narrative; and a lack of social interaction burden around receiving the narrative [5]. The same study presented a change model in which impact begins with the recipient connecting to events in the narrative or to characteristics of the narrator. Impact was reduced if the recipient was experiencing a crisis, and was positively moderated by the perceived authenticity of the narrative. Receiving recovery narratives created cognitive and affective change in perceptions of connectedness, validation, hope and optimism, empowerment, appreciation, reference shift and reduction in self-stigma. The definition of appreciation encompassed a subset of experiences identified as “meaning in life” in a systematic review on recovery processes [46]. Feeling empowered led to helpful behavioural changes emulating those of the narrator, such as increased likelihood of disclosure of mental health experiences to others and greater ability to exert control during interactions with mental health workers. Harmful transdiagnostic forms of cognitive and affective change can also be created by receiving recovery narratives. These include perceptions of inadequacy, disconnection, pessimism and burden. Interventions utilising recovery narratives should consider how to manage and ameliorate harmful change [5].

A recent qualitative study [47] has refined the mechanism of connection presented in [46]. It has identified three factors underpinning connection: comparison of self to narrator or narrative; feeling empathy for the narrator; and learning something from the narrative.

A recent systematic review [4] provides additional specific items of knowledge that complement these two qualitative studies, which post-date the review. It found that recent traumatic events disrupt connection to a narrator or narrative and hence reduce potential impact. Receiving the recovery narratives of people experiencing eating disorders can cause diagnostically specific harmful behavioural responses in those with prior experience of eating disorders, in the form of emulating harmful behaviours described by a narrator, especially if the matched behaviours had been previously enacted by a recipient. Emulation of narrator behaviours was initiated by the elements of eating disorder recovery narratives that described adversity or struggle. It was potentiated by any specific detail about eating disorder behaviours taking place during these periods, such as narrator estimates of how many calories they were consuming.

The preceding evidence is primarily transdiagnostic, since recovery is a multicomponent process which is not diagnosis-specific [46]. However, there is specific evidence that indicates possible benefits of recorded recovery narratives in relation to people living with psychosis. An Australian study identified benefits from recorded recovery narratives in three domains: being inspired; knowing I’m not alone; and believing recovery is possible [34]. Recovery narratives can create hope, and messages that create hope are known to be recovery-promoting in psychosis [48]. Feeling more hopeful can also support recovery through re-imagining the self [49], and hope mediates potential psychosis recovery indicators such as increases in structured activity [50]. People experiencing psychosis regularly use digital technologies such as social networks [51]. Furthermore, a systematic review of interventions for psychosis incorporating online, social media and mobile technologies concluded that these approaches are acceptable, feasible and have the potential to improve outcome [52].

No prior randomised controlled trial (RCT) on the use of recorded recovery narratives to provide benefits for people experiencing psychosis has been conducted, and an RCT would inform the development of diagnostically specific clinical guidelines for the use of recovery narratives with this population. We will conduct a definitive pragmatic [53] RCT, the *Narrative Experiences Online (NEON) Trial*, which incorporates an economic and process evaluation. Recovery narratives and all trial procedures (including randomisation) will be delivered online through the NEON Intervention, a non-medical online interface designed with the intent of supporting people experiencing a wide range of mental health problems. The NEON Intervention provides a variety of mechanisms for accessing the NEON Collection of recovery narratives. These include the use of a hybrid recommender system [54], which uses both collaborative filtering [55] and content-based filtering [56] to generate automated recommendations of recovery narratives, tailored to information collected about participants. The content-based portion of the recommender system uses a model trained using supervised machine learning [57] to identify content that might provide benefits for a user.

In addition to people living with mental health problems, recovery narratives may be relevant to their informal carers, such as family members, friends, neighbours and other unpaid supporters. Many carers struggle with feeling pessimistic about the possibility of recovery for their loved ones [58], and there is evidence that being more “recovery-aware” gives informal carers more hope and optimism about the future [59]. Established recovery frameworks are also relevant to the experiences of informal carers, supporting processes such as maintaining hope, reconnecting, overcoming secondary trauma and (for family members) journeying from carer to family [60]. Although the knowledge base is less developed than for people with mental health problems, current evidence suggests that recovery narratives may also be beneficial to informal carers. As such, we will use the same digital infrastructure to conduct an exploratory study of the use of the NEON Intervention for informal carers (the *NEON-C Trial*), to inform the design of a future definitive RCT. Given the transdiagnostic benefits of recovery narratives previously identified, we will also run a second exploratory study with people with non-psychosis mental health problems (the *NEON-O Trial*).

### Study aims and objectives

#### NEON Trial

The aim of the NEON Trial is to understand whether receiving online recorded recovery narratives benefits people with experience of psychosis.

The NEON Trial has primary and secondary objectives.The primary objective is to evaluate the effectiveness of the NEON Intervention in improving quality of life at 1 year follow-up.

The primary hypothesis is that, compared to control group participants not receiving the NEON Intervention during that year, intervention group participants who receive the NEON Intervention will have a clinically important increase in quality of life 1 year later. Control group participants will continue to receive usual care, which has been described as the “comparator of choice” (p. 92) [61] for pragmatic trials.

The secondary objectives are:

1. To evaluate effectiveness in improving hope, empowerment and meaning in life and in reducing symptomatology
2. To evaluate the cost-effectiveness of the intervention compared with treatment as usual, from both a health and social care provider and a societal perspective
3. To understand how the intervention is used and experienced
4. To evaluate the trial change model
5. To evaluate the performance of the supervised machine learning algorithm in producing a model that matches recovery narrative content to participants
6. To understand how the model trained by the machine learning algorithm develops through the trial
7. To determine whether the effectiveness of the NEON Intervention varies according to prior health service usage by a participant.

The trial also has exploratory objectives:

1. To identify potential predictors of outcome, to inform the design and analysis of future trials
2. To examine how the effect of the intervention varies over time and by dose.

#### NEON-O and NEON-C trials

The aim of both exploratory trials (NEON-O and NEON-C) is to develop knowledge to support the design of a future definitive trial with the target population.

The objectives are:

1. To optimise the intervention to the target population, by using usage data to understand patterns of dose and adherence, in order to identify candidate refinements to the intervention
2. To optimise the evaluation to the target population, including informing the choice of primary and secondary outcome measures in a future trial
3. To establish trial parameters relating to the target population, by evaluating recruitment procedures, estimating recruitment rates and making a preliminary estimate of effect size to inform a future power calculation
4. To evaluate the performance of the supervised machine learning algorithm in producing a model that matches recovery narrative content to participants
5. To understand how the model trained by the machine learning algorithm develops through the trial
6. To understand the acceptability of the intervention to the target population.

The design decisions outlined in this protocol have been optimised for the NEON Trial. Aspects of design which differ in NEON-O and NEON-C are identified.

### Study framework for evaluation

The Evidence Standards Framework for Digital Health Technologies [62] has been used as a guiding framework for evaluating the effectiveness of the NEON Intervention. Within this framework, the NEON Intervention is categorised as a tier 3a DHT, intended to enable preventative behaviour change or allow self-management of a diagnosed condition. A feasibility study has provided observational evidence required for tier 3a DHTs (Slade, Rennick-Egglestone, Llewellyn-Beardsley et al: Using recorded mental health recovery narratives as a resource for others: Narrative Experiences Online (NEON) intervention development, submitted). All other evidential requirements are covered by this trial protocol.

### Study change model for the impact of recorded recovery narratives

**Fig. 1:**
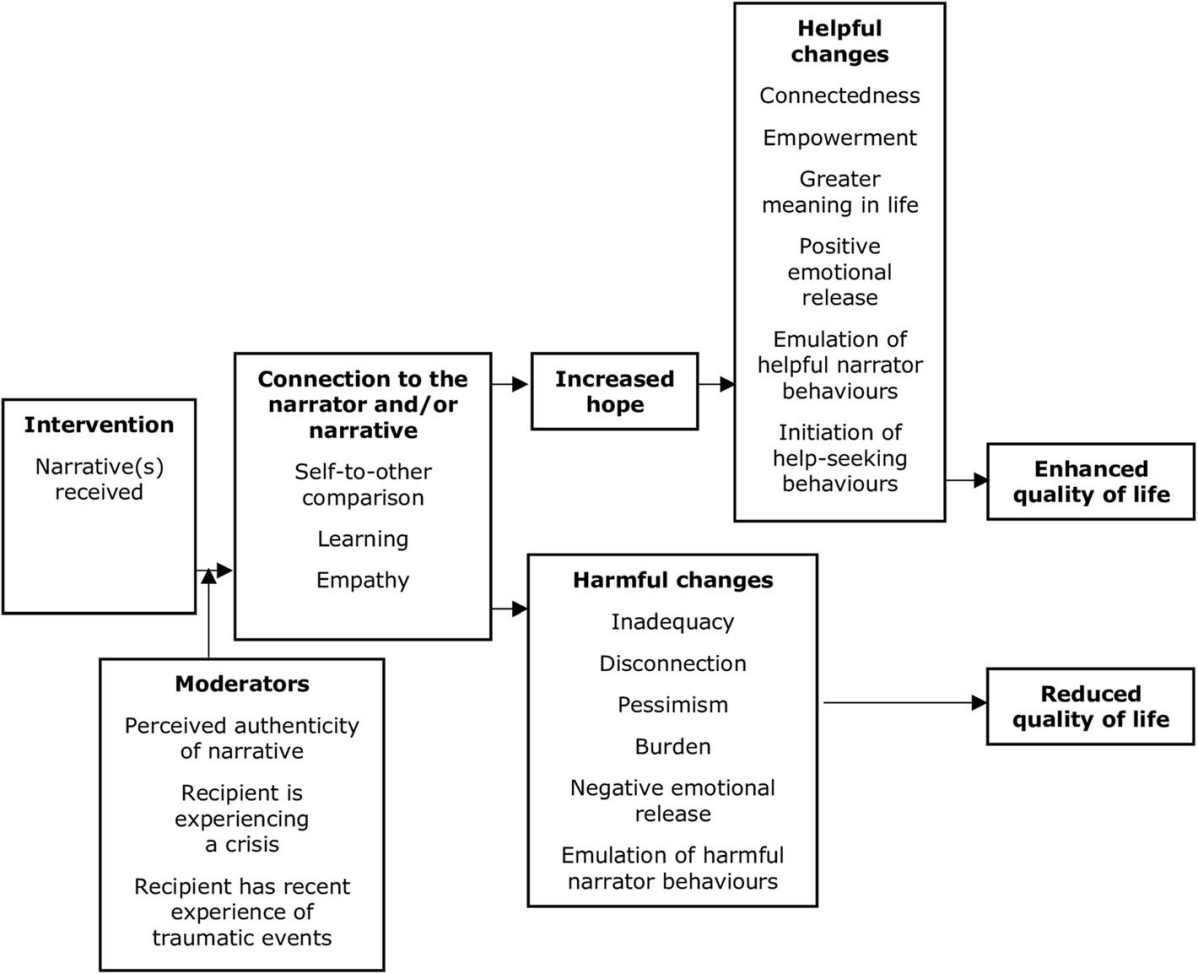
NEON change model

A change model has been synthesised from frameworks developed in a systematic review [4] and qualitative study [5]. The most empirically supported elements of these frameworks were integrated, with priority given to those which can be evaluated in a clinical trial with a process evaluation. A specific focus was on the causal chain of intermediate mechanisms between intervention and outcome. The change model contains no diagnostically specific elements and hence is appropriate for use in all three trials described in this protocol. The change model is presented in Fig. 1.

Initiation of help-seeking behaviours is included as a helpful change, due to evidence that this can generally be produced through exposure to mental health material used in anti-stigma campaigns [30], although no evidence as yet links initiation of help-seeking behaviours to receiving recovery narratives specifically.

The change model includes *emulation of harmful behaviours* as a general form of harmful change caused by receiving recovery narratives. Whilst existing research evidence for this is limited to recipients with prior experience of eating disorders, receiving online material featuring self-harm is known to have the capacity to potentiate self-harm [63], and inclusion of a more general formulation of harmful behavioural change in the change model enables the selection of mechanisms to manage it. As such, this inclusion is justifiable on the biomedical principle of non-maleficence [64].

## Methods

The NEON Trial is an RCT with an internal pilot and an economic and process evaluation, and with all study procedures other than process evaluation interviews conducted online. The internal pilot sample will comprise participants recruited during the first 3 months of the trial, with trial recruiting continuing thereafter. NEON Trial participants who meet the inclusion criteria will be individually randomised into one of two treatment groups (control group, intervention group) with an allocation ratio of 1:1.

Follow-up is at 1 week, 12 weeks and 52 weeks after randomisation, with the primary endpoint at 52 weeks. The cost-effectiveness of the NEON Intervention will be established by calculating the costs of delivering the NEON Intervention, the impact on services costs of receiving the intervention and the change in quality-adjusted life years (QALYs) due to receiving the intervention.

The NEON-C and NEON-O exploratory trials are RCTs with a limited process evaluation. Participants who meet the inclusion criteria will be individually randomised into one of two treatment groups (control group, intervention group) with an allocation ratio of 1:1. The same outcome data will be collected as for the NEON Trial, at the same timepoints, but only exploratory clinical and economic analyses will be conducted. As for the NEON Trial, all study procedures other than process evaluation interviews are conducted online. Up to 20 semi-structured interviews will be conducted for the process evaluation in each of the NEON-C and NEON-O trials.

Participants will not be blinded to allocation status in any of the three NEON trials. There will be no exclusions based on current treatment.

**Fig. 2:**
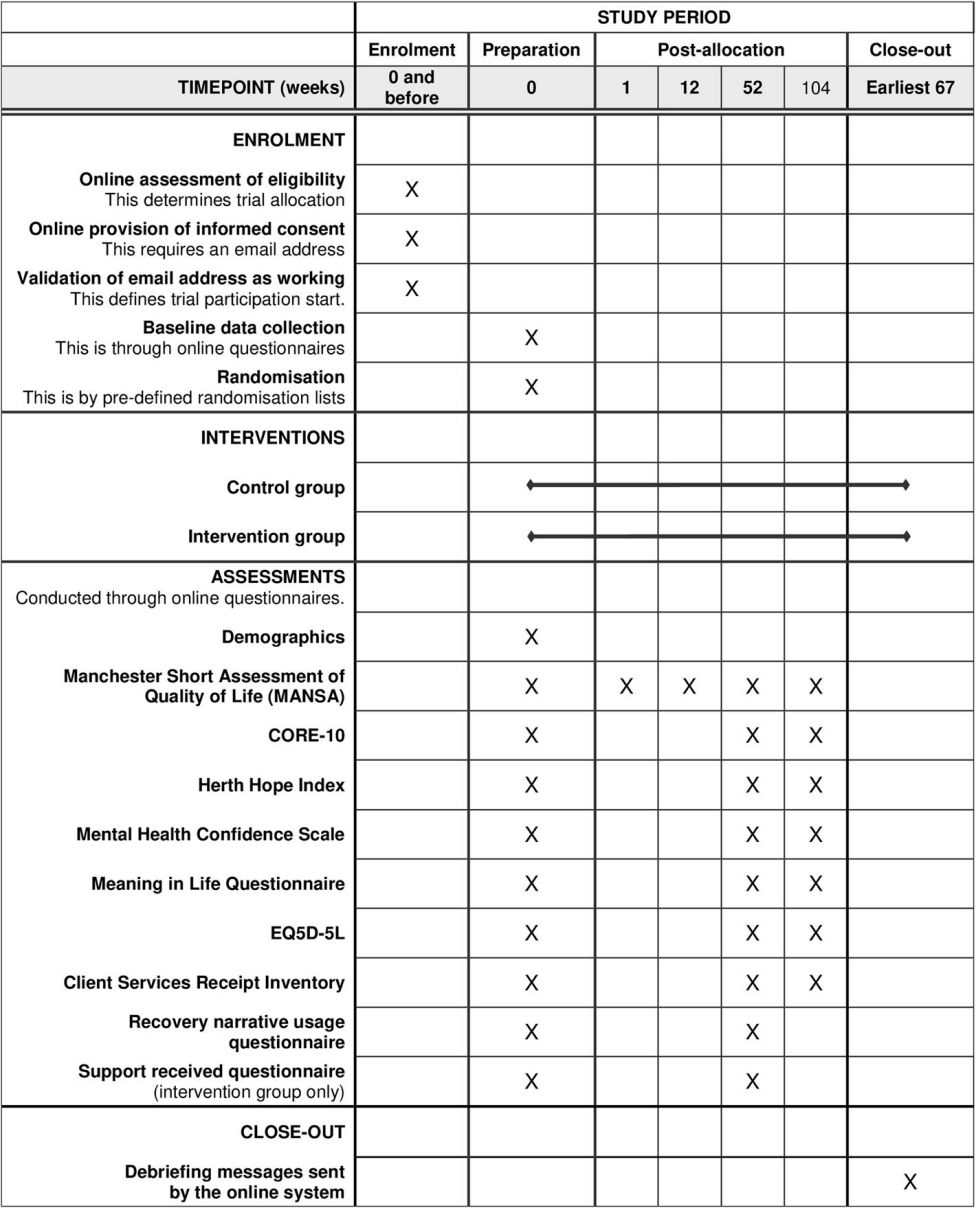
Schedule of enrolment, interventions and assessments for all three trials

The schedule of enrolment activities, interventions and assessments is shown in Fig. 2.

Assessments at 1, 12 and 52 weeks are required for clinical and economic analyses. The assessment at 104 weeks is not required, as only early recruits will reach this before the study end date. Participation in interviews for the internal pilot and process evaluation is optional and not included in the figure.

### Population

The study populations for the three trials are defined in the following sections. All are self-rated, using a shared online interface. Details are provided in the study procedure on Eligibility. No formal thresholds will be applied for language comprehension.

Participants will only be allowed to take part in one of the trials. Where participants meet the inclusion criteria for more than one trial, exclusion criteria have been included to specify that the order of preference is NEON Trial followed by NEON-O Trial followed by NEON-C Trial.

#### The NEON Trial

The inclusion criteria for the NEON Trial are as follows:

1. Experience of psychosis in the last 5 years
2. Experience of mental health-related distress in previous 6 months
3. Resident in England
4. Aged 18 or older
5. Capable of accessing or being supported to access the Internet, either on a personal computer, mobile device or at a community venue
6. Able to understand written and spoken English
7. Capable of providing online informed consent.

#### The NEON-O exploratory trial

The inclusion criteria are:

1. Experience of mental health problem other than psychosis in the last 5 years
2. Experience of mental health-related distress in previous 6 months
3. Resident in England
4. Aged 18 or older
5. Capable of accessing or being supported to access the Internet, either on a personal computer, mobile device or at a community venue
6. Able to understand written and spoken English
7. Capable of providing online informed consent.

The exclusion criterion is:

1. Eligibility for the NEON Trial.

#### The NEON-C exploratory trial

The inclusion criteria are:

1. Experience of being an informal carer for someone with experience of mental health problems within the last 5 years
2. Resident in England
3. Aged 18 or older
4. Capable of accessing or being supported to access the Internet, either on a personal computer, mobile device or at a community venue
5. Able to understand written and spoken English
6. Capable of providing online informed consent.

The exclusion criteria are:

1. Eligibility for the NEON Trial
2. Eligibility for the NEON-O Trial.

### Interventions

#### Control group

In all three trials, participants allocated to the control group will have no changes to any treatment they may be receiving.

For the NEON Trial and NEON-O Trial, participants will include:

1. People currently receiving no mental health treatment
2. People receiving primary care mental health treatment, such as pharmacotherapy from their family doctor/general practitioner (GP) or counselling from a primary care counsellor
3. People receiving support from the Improving Access to Psychological Therapies (IAPT) programme, which provides evidence-based psychological therapies and routine outcome monitoring to people living with common mental disorders such as anxiety and depression, with an increasing availability of services for people living with psychosis and other severe mental illnesses [65]
4. People receiving treatment from secondary mental health services, such as locality-based mental health teams or hospital-based services. In secondary care, treatment typically involves multidisciplinary care coordination under the Care Programme Approach [66], a national framework for care coordination and resource allocation in mental healthcare whose key features include systematic arrangements for assessing health and social needs; formation of a care plan identifying the health and social care required from a variety of providers; appointment of a key worker to monitor and coordinate care; and regular review of the care plan.

For the NEON-C Trial, participants will not currently be experiencing mental health problems, as otherwise they would be eligible to participate in the NEON Trial or NEON-O Trial.

Participants allocated to the control group in all three trials will receive access to the NEON Intervention after 52 weeks, for at least 1 month or until the trial closes, whichever is later. During this period, logging data will be collected on their usage of the intervention.

#### Intervention group

For all three trials, participants randomised to the intervention group will continue to receive their usual care (if any). Typical offerings are as described for the control group. The intervention group will also receive immediate access to the NEON Intervention.

The NEON Intervention is a password-controlled, online interface which presents mental health recovery narratives either sourced from existing public collections such as books, health service booklets and online collections, or donated specifically to the NEON study by individuals. Narratives are managed in line with a protocol previously approved by the Health Research Authority (HRA) (Integrated Research Application System [IRAS] 247343, Research Ethics Committee [REC] reference 18/LO/0991).

The NEON Intervention is accessed through a web browser, either on a mobile phone or on a laptop or desktop computer. It provides four routes to accessing recovery narratives, which are described in the following paragraphs, one of which uses an algorithm to match narratives to participants. This is referred to as the *matching algorithm* in the remainder of this protocol. Information about participants used to generate matches is referred to as *matching data* and is stored in a *personal profile* along with other forms of personal information needed by the NEON Intervention. Information stored in the personal profile is detailed in Additional file 1. All items in the personal profile are considered to be research data. Titles or categories used to display personal profile contents to participants may be updated (for example, in response to feedback collected through the internal pilot).

The NEON study Lived Experience Advisory Panel (LEAP), consisting of 10 members with personal experience of mental health problems, have advised that participants should be able to provide as little or as much information in their personal profile as they wish, and hence we have minimised mandatory items in the personal profile. Although there is some overlap with the demographics form used by the NEON trials, the contents of the personal profile are not auto-populated from the demographics form. This maintains a separation between trial procedures and intervention usage. The exception is contact details provided through the consent form, which are essential for operation of the NEON Intervention. Here, the personal profile will be auto-populated to reduce participant burden.

After signing in to the NEON Intervention for the first time, the participant is sequentially shown a number of introductory pages intended to facilitate learning how to work with the NEON Intervention, and to collect enough information for the NEON Intervention to function effectively. These pages will not appear on subsequent logins. First interactions with a mental health technology are known to present particular difficulties for users experiencing mental health problems [67]; hence, these pages have been designed to help a new user rapidly acclimatise to the NEON Intervention.

The introductory pages appear in the following sequence:

##### “Welcome” page

This page provides a brief overview of how to use the NEON Intervention; seeks to normalise emotional responses to recovery narratives; and provides initial guidance on how to deal with difficult emotional responses.

##### “Initial information” page

The Initial information page allows the participant to provide an initial set of entries for all “directly editable” items in their personal profile (see Additional file 1). To support participants in managing their own safety, this includes a list of types of narrative content that they wish to hide, using a typology of content warnings developed by the NEON study.

Some participants will be experiencing conditions that disrupt processing of particular formats of narrative, e.g. text-based narratives in the case of dyslexia. Some participants may have to use public computers to access the NEON Intervention and hence may wish to avoid formats of narrative that include audio. As such, users can select formats of narrative that they do not wish to receive. The NEON Intervention interface will not allow users to block all formats, as then they would not be able to receive any narratives.

The Initial information page will include text indicating that personal profile contents can always be updated through the “About Me” button during future usage of the intervention.

##### “First story” page

This provides a first experience of receiving a short narrative, so that the user experiences this as early as possible in usage of the intervention. A short narrative will be displayed on this page. Only narratives that do not have content warnings will be considered in scope for selection so as to minimise chances of distress. The selected narrative will not be of a format blocked by the user, and hence some users will receive different “first stories”.

After receiving this narrative, the participant will be asked to rate it for hope, and optionally four types of connection mechanisms. The following questions and anchor points will be used, with indicated questions, numbers and numerical ranges *not* visible to participants.

(Mandatory)

Q1: How hopeful did the story leave you feeling? [range –1 to 2]

Less hopeful than before - No change - A bit more hopeful - Much more hopeful

(Optional)

Q2: How similar was the story-teller to you? [range 0 to 3]

Not at all - A bit - Quite a lot - Very much

Q3: How similar was the story-teller’s life to your life? [range 0 to 3]

Not at all - A bit - Quite a lot - Very much

Q4: How much did you learn from the story? [range 0 to 3]

Nothing - A bit - Quite a lot – A huge amount

Q5: How emotionally connected did you feel with the story? [range 0 to 3]

Not at all - A bit - Quite a lot – A huge amount

Q2 and Q3 have been selected to operationalise the connection mechanism referred to as “Self-to-other comparison” in the trial change model (Fig. 1). Q4 operationalises the connection mechanism referred to as “learning”. Q5 operationalises the connection mechanism referred to as “empathy”. Responses to these five questions are referred to as *narrative feedback* in the remainder of this protocol, and will be used as matching data. The NEON Intervention will encourage participants to provide narrative feedback after each narrative received through usage of the NEON Intervention, although it is not technically possible to enforce this, since participants can always close their web browser if they do not wish to provide feedback.

The pool of narratives considered in scope for usage as the first story will be reviewed approximately every 3 months after trial start. Drawing on all narrative feedback provided by trial participants up to that point, a small number of narratives will be selected which have received hope ratings with a high mean and small standard deviation (SD), as these are most likely to be beneficial.

LEAP have advised that participants should be able to *block* any story at any point (e.g. even partway through reading or watching it), for example, if they found it excessively distressing. LEAP have also advised that recipients should be able to bookmark a story, e.g. to allow an influential story to be re-visited or discussed with a support worker. As such, buttons to block and bookmark stories will be provided on the same screen as the first story, and for all other subsequently accessed stories.

After viewing the first story and providing narrative feedback, the participant is given access to the intervention home page. This presents four buttons in an ordered list, allowing participants to access recovery narratives in different ways:

- *“Match me to a story (recommended)” button*. Requests the automated recommendation of narratives matched to the participant, presented as a list of stories. This will be the recommended approach to narrative selection; hence, it appears first in the list. The participant can choose to receive just one narrative or can examine all in the list. The list will only include narratives not seen before.
- *“Get me a random story” button*. Requests a randomly selected narrative that the user has not seen before, using an algorithmic pseudo-random number generator.
- *“Browse stories” button*. Shows available narratives grouped by tags, so that the participant can browse them. For example, the database may contain 245 narratives which relate to employment. The participant can narrow the search by selecting multiple tags, and can choose from narratives matching selected tags.
- *“My stories” button*. Shows a list of recovery narratives previously received, unless they have been blocked, in which case they will not appear. They are presented in two groups: (1) narratives previously bookmarked by the participant, (2) hopeful stories (those rated highest for hope, either as indicated by the participant or by the cohort as a whole). The participant can select a bookmarked or hopeful narrative to be re-received.

The home page also contains a button labelled *“About Me”*. Clicking this button opens a page allowing the participant to update any information in their personal profile marked as “directly editable” in Additional file 1. It contains a link to a safety event reporting form, in case the participant has experienced a serious adverse event (SAE), and also a function to allow participants to unblock all blocked narratives. Since even the titles of narratives might be distressing in some circumstances, this function will not display a list of all narratives that have been blocked, and will instead just summarise the number of blocked narratives.

To enable easy navigation, the footer of the NEON Intervention, which is always available regardless of which page is selected, will contain five buttons: Home, Welcome, About NEON, I’m upset, Get me out of here.

Clicking these buttons causes the following actions:

- *“Home” button*. Takes the user straight to the intervention home page.
- *“Welcome” button*. Displays information previously provided on the “Welcome” and “Useful Information” pages.
- *“About NEON” button*. Opens a page giving more detailed information about the NEON Intervention, including aims, how narratives were collected, how to make best use of the intervention, information about the funders, information about the study team (including a link to the study website http://researchintorecovery.com/neon), functionality to view the consent form and functionality to initiate a withdrawal from the trial.
- *“I’m upset” button*. Opens a page giving information about dealing with difficult emotional responses. This will remind participants of any self-management strategies they have identified. It will suggest common self-management strategies that might help them. It will provide links to organisations and services that can be accessed by participants, including charities and statutory health services. The design of this page has been refined with LEAP.
- *“Get me out of here” button*. Clicking this button immediately closes the NEON Intervention web page and logs the user out of the NEON Intervention. It immediately takes the user to a neutral web page (http://www.google.co.uk).

To distinguish the NEON Intervention from processes associated with the trial (e.g. information sheets, completion of measures), the NEON Intervention will not be branded with study sponsor or research site logos, and it will be presented with a contrasting colour scheme. This is to support the ecological validity of the evaluation by creating a visual boundary between trial procedures and intervention content.

Participants can use the NEON Intervention as little or as frequently as they wish, and there is no expected pattern of usage. Patterns of usage will be monitored algorithmically. If the participant has not used the intervention for 1 month, then a reminder message (which can be opted out of) will be sent through contact mechanisms specified on the “About Me” page. This will encourage the participant to re-visit the intervention and give an option to access online information about dealing with technical problems, such as reminders about the login procedure. Messages may also be sent when new narratives that might be of interest to participants are added, depending on the frequency of narratives being added to the database.

### Measures

All measures are included in Additional file 2. All outcome measures to be used in the clinical outcomes analysis are summarised in Table 1. The same measures and timepoints will be used in all three trials. Responses to items will be collected online, and validation rules incorporated into online forms will ensure no missing items.

**Table 1:** Outcome measures used in the clinical outcomes analysis. “x” indicates a timepoint where measures are collected; bold text indicates primary endpoint

Domain	Measure	Items	Report	Timepoint (week)			
				0	1	12	52
Quality of life	Manchester Short Assessment of Quality of Life	12	Mean item score Range 1–7 Higher better	x	x	x	**x**
Symptomatology	CORE-10	10	Total item score Range 0–40 Lower better	x			**x**
Hope	Herth Hope Index	12	Total item score Range 4–48 Higher better	x			x
Empowerment	Mental Health Confidence Scale	16	Total item score Range 16–96 Higher better	x			**x**
Meaning in life	Meaning in Life Questionnaire	10	Mean item score for *presence* and *search* subscales Range 1–7 Higher better	x			**x**
Total: 60 items							

The primary outcome measure used in all three trials is quality of life, assessed with the Manchester Short Assessment of Quality of Life (MANSA) [68] at baseline and all follow-ups. MANSA has been successfully used to assess quality of life in individuals with psychotic disorders [69, 70] and other forms of mental health problem [71]. The score for MANSA is calculated from the 12 subjective items in Section 3 of the measure [68].

Four clinical secondary outcome measures are used in the three trials. The CORE-10 is a self-rated measure of mental health distress, which includes 10 items relating to depression, anxiety, trauma, functioning and risk to self [72]. The Herth Hope Index is a 12-item self-rated abbreviated version of the Herth Hope Scale [73]. The Mental Health Confidence Scale is a self-rated measure of self-efficacy amongst persons dealing with mental disorders [74]. The Meaning in Life Questionnaire is a 10-item measure incorporating two subscales: presence of meaning in life, anddegree of search for meaning in life [75]. All secondary outcome measures have been used successfully with individuals experiencing psychotic disorders [65, 76–78].

Two measures are included for use in the health economics analysis for the NEON Trial. The EQ-5D-5 L [79] is a five-item self-completed measure of health-related quality of life which is used across a broad range of health conditions. The Client Service Receipt Inventory (CSRI) is a measure of service use that enables service costs to be estimated and which can be tailored to each study’s requirements [80]. A version of the CSRI has been produced which collects service use data covering primary care, secondary mental and physical care, social care and time away from usual activity/employment, defined using employment categories presented in the genetic mental health version of the full CSRI [81]. These have been selected as the major cost drivers of provision for the NEON Trial population. Item count has been abridged relative to a typical item count for the CSRI so as to limit the total burden on participants of completing measures. CSRI completion at baseline will have a 6-month retrospective period, and CSRI completion at 52 weeks will have a 12-month retrospective period. The same data is collected in the NEON-O and NEON-C trials. The health economics measures are summarised in Table 2. Opportunistically, the same follow-up data will be collected at 104 weeks for intervention group participants who reach this timepoint, to allow for exploratory analysis of the longer-term impact of receiving the intervention. Eligible participants will be those who are randomised to the intervention group before end of April 2020.

**Table 2:** Health economics measures. “x” indicates a timepoint where measures are collected

Domain	Measure	Items	Timepoint (week)			
			0	1	12	52
Health-related quality of life	EQ-5D-5 L	5	x			x
Service use	Client Service Receipt Inventory (abridged). 6-month retrospective at baseline, 12 months retrospective at primary endpoint	10	x			x

### Power calculation

The NEON Trial is powered on mean item score for MANSA. The primary endpoint for the NEON Trial is a minimally clinically important difference in mean item score. This is defined as an improvement of 1 scale point in 3 out of 12 items at 1 year follow-up in the intervention group relative to the control group. A total sample size of 683 (approximately 341 participants per arm) will provide 90% power to detect a minimally clinically important effect size (Cohen’s *d*) of 0.27, allowing for 20% attrition (SD = 0.9 [82], power = 0.9, *p* = 0.05). This will give an analysable sample of 546 (273 participants per arm).

The sample sizes for the NEON-C and NEON-O trials have been chosen in order to calculate preliminary effect size estimates to inform power calculations for future trials. A total pilot study sample size of at least 70 has been recommended to estimate the standard deviation of a continuous outcome with good precision [83]. This general rule has also been shown to be sufficient in minimising the overall sample size across the pilot and main trial when medium effect sizes are expected [84]. Allowing for 20% attrition, the target sample size for both NEON-C and NEON -O will be at least 88 (44 per arm). We have decided to use a conservative rounded-up sample size of at least 100 (50 per arm) to reflect possible uncertainty in the attrition level.

### Procedures

#### Recruitment

The planned recruitment period for all three trials is 14 months. The mean recruitment rate for the NEON Trial is 49 participants per month.

Effectiveness studies evaluate treatments in “real-world” conditions [85]. An analysis of community survey data from 18 countries found that mean lifetime prevalence of ever having a psychotic episode was 5.8% [86], whilst an epidemiological study conducted on a US community sample estimated lifetime rates of psychosis service usage in a range from 0.2% (narrowly defined criteria) to 0.7% (broadly defined) [87]. Although these studies cover different populations, we have assumed for the purposes of recruitment planning that there is a substantial population of people with experience of psychosis but no engagement with statutory services. Recruitment strategies for the NEON Trial will be designed to target a purposive sample [88] of the target population, with the sample containing a representative spread of experiences of health service support for psychosis experiences. Informed by the epidemiological evidence, this will include participants who have received no support from health services. The same recruitment methods will be used for all three trials, but recruitment effort will be prioritised to the NEON Trial, which has the largest target sample size.

The following recruitment methods will be used to make potential participants aware of the three NEON trials: online advertising (disseminated on the study website, by email and through social media networks); advertising in print media; placement of posters and leaflets in health service and community venues and in public places; snowball recruitment; recommendation by general practitioners, mental health workers and social workers to clients (either in person or by other communication mechanisms legitimately used by these practitioners); direct approach by researchers to individuals who might be interested in the study (either in person or by other communication mechanisms legitimately used by researchers to contact potential participants); presentations by the study team; appearances of the study team in national media; and recommendation by public figures with an interest in mental health. Where individuals are to be approached directly, governance of what is considered a legitimate approach will be delegated to research sites. For example, some research sites will have systems in place which allow for the management of “consent to contact” lists. These can be used to approach potential participants in the three NEON trials if they are authorised for use for these trials at the research site.

Where promotional material is used, it will vary greatly in length and amount of information, e.g. between text used in tweets and text used in posters. We would anticipate sending out at least 100 pieces of promotional material, each tailored to a different audience and to the current state of the trial. Early on, we may send out broadly relevant messages, and later we may send out messages that are more targeted at under-represented groups.

Principles to inform the text for all advertising are given in Additional file 3. These principles allow for the generation of recruitment material that is coherent and ethically sound, but which can also be updated as our understanding of how to promote the trial develops, for example, in response to the analysis of the NEON Trial internal pilot. All recruitment material generated will conform to these principles. All promotional material will be logged into the Trial Master File (TMF), with date and location of use, to enable the study sponsor to audit it against the advertising principles.

Sample recruitment posters are included in Additional files 4, 5, 6 and 7. Their graphic design will be updated if necessary, and new graphic designs will be submitted to the HRA as a non-substantial amendment. Posters will not be localised to research sites.

All recruitment activity will result in a participant receiving the web address of the *splash page* for the NEON trials. This is a publicly available online interface which can be accessed from a public or private computer or from a mobile device. The splash page incorporates a link to a login screen for participants who have already enrolled and have created an online account (“If you have a login click here”). It will have a link to a *trial information page* introducing the NEON Trial, NEON-O Trial and NEON-C Trial. This will describe the purpose of the trials and explain the process of enrolling, which may not be familiar to some potential participants (“If you are new to NEON click here”). It will link to a page to allow people to report safety issues (see the section on “Safety event monitoring procedures” for details).

The trial information page will indicate if any trial has closed due to attaining the required participant count. From the trial information page, a potential participant can access an eligibility checking interface. The link to the eligibility checking interface will be removed once all trials have closed for recruitment, and all recruitment relating to that trial will be withdrawn as soon as possible after trial closure.

#### Eligibility checking

To avoid the burden of an ineligible participant engaging in informed consent procedures, potential participants will be asked to answer a short series of questions presented in an online interface. The primary purpose of this interface is to establish eligibility for any of the three NEON trials. The interface will also capture how the potential participant learned about the NEON trials so as to evaluate the effectiveness of different recruitment methods. It will also capture sufficient information to allocate the potential participant to a research site if he/she is found to be eligible for a trial and then choose to complete consent procedures.

For all three trials, the benefits of clinician rating of eligibility are outweighed by the significant extra burden on the participant, the likely lower recruitment rate that would result (as some potential participants would not wish their clinical team to be contacted) and the fact that many potential participants will not be in contact with mental health services.

The interface used to present online questions will be publicly available. No online account is required to access it. No personal data will be stored as a result of interacting with it, as potential participants have not given consent at this point in the study procedures. Anonymous non-personal data will be stored to enable accurate reporting of trial recruitment processes and to inform advertising strategies. Before being presented with questions, potential participants will be shown a message, presented in text, which describes the purpose of the chosen questions and which indicates that potential participants should only fill them out if they are interested in taking part in one of the clinical trials. Carefully crafted instructions can shape online experience and can support compliance with a designer’s intended use for those experiences [89]. The current text to be used is included in Additional file 8. If needed to support effective use by participants, the text of all messages referenced in this protocol will be refined over time, for example, based on feedback collected during the internal pilot.

##### Eligibility checking and recruitment logging questions

Whilst all three trials remain open, questions used to assess eligibility and log information about the recruitment process are shown in Table 3.

**Table 3:** Online questions used to establish eligibility and log information about recruitment

Question	Eligibility decision and next question
Q1: How did you find out about the NEON trials? [Through my family doctor or GP surgery, Through a hospital or mental health service, Other]	Through a hospital or mental health service: go to Q2 All other options: go to Q3
Q2: Was this through any of the following trusts? [List of current secondary care research sites, None of these]	Go to Q3
Q3: Are you 18 or over today, and normally resident in England? [Yes/No]	Yes: go to question Q4 No: not eligible for any trial
Q4: Can you understand written and spoken English?	Yes: go to Q5 No: not eligible for any trial
Q5: Within the last 6 months, have you had mental health problems that: a. Make it hard to manage the day-to-day demands of life? (No, A bit, Yes) b. Currently cause you emotional distress? (No, A bit, Yes) c. Cause you social problems like loneliness? (No, A bit, Yes)	No to all subquestions: go to Q7 Otherwise: go to Q6
Q6: In the last 5 years have you had experiences diagnosed as psychosis, or that you or others would call psychosis (such as seeing or hearing things that others have not, or having unusual beliefs that other people disagree with)? [Yes/No]	Yes: eligible for NEON Trial No: eligible for the NEON-O trial
Q7: Within the last 5 years, have you cared for someone with experience of mental health problems?	Yes: go to Q8 No: not eligible for any trial
Q8: Was this as part of your employment or profession?	Yes: not eligible for any trial No: eligible for the NEON-C trial

Questions 3 through 8 in this table have been discussed with LEAP, and the text of these questions has been updated according to their recommendations. Questions that relate to mental health have been designed to be accessible to people who have never received a formal diagnosis of any mental health condition.

The flow of questions in the eligibility checking interface will change as trials are closed for recruitment; e.g. if the NEON-C trial had recruited all needed participants, then questions 7 and 8 would be removed. In that circumstance, if a potential participant answered no to all items in question 5, they would then be given a message indicating they were ineligible for any trial.

Ability to engage with the eligibility checking interface will be taken as evidence that the potential participant is capable of using an online intervention, either supported or unsupported. Items used in Q5 were drawn from the Threshold Assessment Grid (TAG), a staff-rated measure of the severity of mental illness, for which validity has been established [90]. The phrase used in Q6 for verifying psychosis experiences in potential participants has been developed from an earlier NEON study which successfully recruited 28 participants with experience of psychosis but no formal diagnosis [2, 5, 91].

If a potential participant has entered the eligibility checking interface by clicking on a link in an online advert displayed on a website, the identity of the website displaying the advert will be logged automatically to support an evaluation of recruitment methods, and Q1 and Q2 will be skipped. The potential participant will be allocated to the Nottinghamshire Healthcare National Health Service (NHS) Foundation Trust research site if he/she chose to progress through informed consent procedures, as all participants recruited through non-NHS routes are recruited to this site. To enable this automated process, the web address presented in the online advert will contain a parameter identifying the online system which displayed the advert. As an example, a web address including a parameter of 15 might indicate an advert displayed on the website of the University of Nottingham.

Primary care recruitment for all trials is being managed by primary care teams in the nationwide network of Local Clinical Research Networks (LCRNs). Q1 will enable a reasonable assessment of primary care recruitment success, which will be considered in the analysis of the internal pilot of the NEON Trial.

Secondary care recruitment for all trials is being managed by selected mental health trusts in England, who are operating as research sites. Q1 and Q2 together will enable a reasonable allocation of a participant who learned about the study through secondary care recruitment. If “None of these” is selected for Q2, a potential participant is allocated to the Nottinghamshire Healthcare NHS Foundation Trust if the informed consent procedures have been completed.

If responses to questions indicate that a potential participant is not eligible for any trials, then once the questionnaire has been completed, he/she will be informed of this, through a message designed to reduce the number of people who experiment with responses so as to obtain access to the NEON Intervention. The current text is message 2 in Additional file 8.

If a potential participant is considered eligible for a specific trial, then he/she will next move into informed consent procedures.

#### Informed consent procedures

To ensure that a potential participant is sufficiently informed to provide online consent for participation, an online Participant Information Sheet (PIS) will be provided to people considered eligible to participate in any of the three trials. UK Health Research Authority (HRA) guidance confirms that the online provision of participant information is acceptable [92]. Items in the PIS will be provided in a vertical list, through which participants will be able to scroll up and down. At the end of the PIS, a link will be provided to an Informed Consent Form (ICF). The text/layout for the online PIS is presented in Additional file 9; that for the online ICF is presented in Additional file 10. The PIS will begin with an invitation to take part in a named trial.

For some items, brief text with expandable detail has been provided. This was recommended by LEAP, who reviewed an earlier version of the PIS. It is consistent with emerging evidence that shorter information sheets are more likely to be fully read and more likely to be understood [93], and it exploits the opportunity offered by digital presentation to allow the potential participant to manage how the relevant information is presented. It also takes into account the intrinsically challenging and potentially distressing nature of the first point of interaction with a healthcare technology for a person experiencing mental health problems [67], and is an attempt to make this first contact as accessible as possible. Navigation actions, such as scrolling up and down or opening and closing further information, will be logged anonymously to enable a quantitative evaluation of PIS usage, and the use of expandable details will be explored in the process evaluation. Data collected anonymously will not be linked to the account created for a participant who has completed all consent procedures. The exception will be the research site to which they should be allocated, which is inferred from questions 1 and 2.

The PIS and ICF will contain contact details for the NEON research team. Potential participants will be encouraged to contact the team if they have any questions not answered on the PIS. After reading the PIS, a potential participant will be provided with two buttons, labelled “I *do* wish to take part in the trial” and “I *do not* wish to take part in the trial”. Participant choice will be logged anonymously to allow for accurate reporting of the trial. For participants who do not wish to participate, this message will be displayed:

*Thank you for considering involvement. If you change your mind you are welcome to return and re-register. You can safely close this window.*

Participants who select the “I *do* wish to take part in the trial” button will be asked to complete the online consent form. A joint statement of the HRA and the Medicines and Healthcare products Regulatory Agency (MHRA) on seeking consent by electronic methods [94] indicates that online consent is acceptable for all studies other than Clinical Trials of Investigational Medicinal Products (CTIMPs).

A key advantage of an online intervention is that participants can use a system anonymously if they wish. This feature is particularly relevant to the population for the NEON Trial, since people with psychosis may be particularly vulnerable to concerns about online data usage and may also fear stigmatisation due to mental ill-health [95]. There is evidence that the option to remain anonymous influences decisions about use of online interventions by people with psychosis [96]. The option to remain anonymous has been successfully used in a number of online interventions with this population [97, 98]. Therefore, the person will only be required to check each box on the consent form, rather than providing potentially identifying information such as a signature. This is in keeping with procedures specifically described and allowed in [94]. However, as a minimum, potential participants must provide a valid email address so as to enable the collection of online outcome data. Participants who wish to remain anonymous can use email addresses that do not include their name.

To consent to take part in the study, potential participants must supply all mandatory information required by the ICF, which includes providing a valid email address. They are then provided with two buttons labelled “I agree to take part in the study” or “I do not wish to take part”. If they click “I agree to take part in the study”, they will be given a message indicating that, to complete the registration process, they need to click on a link in a validation email sent to their account. Since a working email address is required for usage of the NEON Intervention, only potential participants who click this link will be enrolled.

After clicking the link, the potential participant is now enrolled in the study. Participants will be asked for a password of their choosing, as it will then be easier to remember. No password complexity rules will be enforced. The participants will be reminded to make a note of the login details and given the option of receiving an automated email or text with the web address and their login details. Although sending such a message constitutes a potential security risk, this is a population who may have cognitive processing and strategic planning deficits. Therefore, the risk in this case is outweighed by the benefits of offering the participants the chance to have all information allowing them to use the intervention in one place.

Participants will not be told of the research site to which they have been allocated. This would be confusing, as once an individual has confirmed participation, all planned participant interactions are either with the NEON Intervention or with the NEON study team.

#### Baseline data collection

At first login, study participants in both groups will be asked to complete baseline measures using an online interface. They will be shown a message which explains the purpose of completing baseline measures; provides an estimate of how long the task will take; reminds them that they can claim a voucher for completing it; and reminds them that measures will need to be completed again later in the trial. Some items in baseline measures include questions that might be perceived as sensitive; hence, the message recommends that the participant should find a private place. The current text is message 4 in Additional file 8. Participants in NEON-O and NEON-C will not be offered any payment for completing measures; hence, for these trials a modified message will be used which excludes information about participant payment.

Participants will then be asked to complete a demographics form and all measures. Each will be presented on a single form, which will start with a title and a single sentence describing the form, to support participant comprehension of purpose. All critical information to include on forms is summarised in Additional file 2. Demographic items on English national ethnicity [99] and on educational attainment [99] have been simplified from those produced by the Government Statistical Service guidance on harmonised questions and concepts for social data sources. An item on recovery status is included for those participants experiencing mental health problems. This incorporates a three-stage model of recovery, which currently has the strongest empirical support [100], including through a study which recruited in England [101].

To minimise data incompleteness, responses will be validated as entered in the online forms used to collect demographics and measures. For example, a participant will not be able to click “Next” until all items on the page have been rated, and can only provide an eligible data value. If the web browser is closed before all items are completed, then participants will be required to continue completion at next logon. After submitting a form, if a participant uses the “Back” button in the web browser, then the form will be displayed again with all data items entered automatically, and the participant will be able to update the values that have been entered and re-submit.

After completing the final form, participants will be given a message thanking them for their responses and confirming once again that their data is confidential. The back button will no longer take them back to a previous form. NEON Trial participants will be provided with a link to claim a £20 voucher as a compensation for the time and effort of completing questions. The voucher will be sent via the participant’s registered email address, using an electronic voucher service provider. Receiving a voucher is optional, and will always be initiated by a participant. A request to be sent a voucher will be logged for study reporting purposes.

In providing payment by voucher, there are two risks to address. One risk is technical error in the implementation of the NEON Intervention, which might result in multiple vouchers being sent to a participant for a single set of completed measures. The second is deliberate fraud, e.g. through a participant registering multiple accounts purely for the reason of claiming multiple vouchers. Thus, the following management strategies have been selected:

1. The PIS will indicate that vouchers are paid up to 1 week after a claim is submitted, to allow the study to team to investigate and verify unusual patterns of voucher claims.
2. Each request for a voucher will require the approval of an administrator, who will match the voucher request to an available code. This means that no voucher codes need to be stored in the NEON Intervention.
3. The Internet Protocol (IP) address of the computer used to make a voucher claim will be monitored. For each timepoint of the study, no more than 10 vouchers per IP address will be paid. This number has been selected to allow for multiple eligible users in the same residence, who might have different logins to the NEON Intervention but share an Internet connection—since domestic routers typically assign the same IP address to all devices connected to the router. It will also account for several individuals accessing the NEON Intervention from the same public computer (e.g. in a public library).
4. Unusual patterns such as more than 5 voucher claims in a single day from an IP address will be investigated by the study team. The study team will contact relevant participants, using their registered email address, to gather information about voucher claims, and will reserve the right to suspend trial participation and to withhold voucher payment if suspicious behaviour is identified. Decisions will be made by the Chief Investigator (CI), with reasons reported to the study sponsor and logged in the TMF. If trial participation is suspended, the participant will not be included in study analyses.

#### Randomisation

Participants will then be randomised to either the intervention group or the control group. The intended allocation ratio (intervention group:control group) is 1:1 for all trials. No stratification of participants on any baseline covariates will be conducted, as existing research does not provide sufficient evidence to reliably identify covariates [102]. Randomisation will be through permuted block randomisation [103] with randomly varying block length. This will use pre-computed lists uploaded by an independent statistician.

Blinding of participants to allocation status is not possible, given the design of the interventions. Control group participants will be given a message reminding them that they are still an important part of the trial, and that they will receive access to the NEON Intervention in 1 year. The current text is message 5 in Additional file 8. Intervention group participants will be told that they will receive immediate access to the NEON Intervention, and asked not to share their login details with others, to reduce contamination. The current text is message 6 in Additional file 8. After receiving this message, intervention group participants are then taken to the “Welcome” page, as described in the previous “Intervention group” section. At future logins, intervention group participants go directly to the intervention home page.

Control group participants are taken to a cut-down version of the intervention home page, which only displays the “About NEON”, “About Me” and “I’m upset” buttons. The About Me button links to a cut-down version of the About Me page, which only allows for the updating of contact information and for participants to open a safety event reporting form.

#### Follow-up data collection

Participants will be asked to complete follow-up measures at the timepoints shown in Tables 1 and 2. A request will be sent using current contact information for the participant, e.g. as collected through the online ICF or updated through the “About Me” page. The request will include a web address that allows the participant to fill out outcome measures. Intervention group participants can also be prompted through the NEON Intervention if they log into it at a timepoint when outcome data can be collected.

When entering follow-up data, the same validation procedures will be used as for baseline data collection, and the same payment procedures will be used (i.e. a £20 voucher will be offered on completion of measures at each follow-up timepoint). Follow-up data will be considered valid if provided within 2 weeks of the 1 week follow-up date, and if provided within 1 month of all other follow-ups. Decisions on how to handle data which falls outside of these windows will be detailed in the statistical analysis plan (SAP). Incomplete forms will remain available up until the start of the next follow-up period. For example, if a participant fails to fill out the week 1 MANSA questionnaire, then the questionnaire will remain available at next login up until the start of the week 12 follow-up period, at which point the participant would receive the week 12 MANSA questionnaire.

At all follow-ups, all participants will be asked to complete a *recovery narrative usage* questionnaire to track contamination. To inform the process and economic evaluation, at 52 weeks follow-up all *intervention group* participants will be asked to complete a *support received* questionnaire. Both are detailed in Additional file 2.

#### Reviewing consent and initiating withdrawal

Participants can view their consent form and initiate withdrawal by logging into their account and viewing the “About NEON” page, which is visible to both control group and intervention group participants. This page contains a button labelled *Consent*, which links to a page providing options labelled (1) look at the consent form and (2) I wish to withdraw from this study. If participants select (2), they are shown a message allowing them to either confirm their request, ask for a discussion with a NEON researcher or cancel their request. The current text is message 7 in Additional file 8. If they choose to withdraw, they receive message 8, which tells them that all identifiable information has been deleted, and tells them how to provide anonymous feedback about the intervention. If they request a discussion, they receive message 9, which tells them how a researcher will get in contact with them. If they choose to cancel their request, they are then taken to the intervention home page.

#### End of study participation

Access to the NEON Intervention must close before the end of the NEON study unless alternative funding arrangements are identified. The end of a period of engagement with a mental health technology needs to be carefully managed, as it has the potential to be an emotionally charged process, especially if the technology has provided benefits to a user [67].

To support participants in the NEON trials through the ending of their engagement with the NEON Intervention, a message will be sent no later than a month before a participant will lose access. This will thank the person for his/her participation and inform the participant of when he/she will lose access (message 10 in Additional file 8).

Once their participation has concluded, participants will be sent message 11 (Additional file 8), which will indicate other sources of recovery narratives that they can consult, using a public list maintained by the NEON study [104] and intended to remain publicly accessible beyond the close of the NEON study.

#### Automated data logging

Logging data will be collected for a range of interactions with NEON interfaces, to support accurate reporting of the trial and for use in the clinical and process evaluations. Only anonymous data will be logged until consent procedures have been completed. Table 4 summarises the events logged about potential or enrolled participants. Each event will be given a unique name to distinguish it in the log files (e.g. ELIGIBILITY_START for the first item in the table).

**Table 4:** Logs of participant system usage to collect

Event to log	Information logged
Potential participants completes first question of eligibility checking process	PPID, DATETIME
Potential participant completes eligibility checking process	PPID, How Found, Research site, Allocated trial, Q5 responses, DATETIME
Potential participant expands or collapses item in the PIS	PPID, PIS item number, PIS item action, Allocated trial, DATETIME
Potential participant navigates up or down through the PIS	PPID, PIS action, Allocated trial, DATETIME
Potential participant makes decision about participation	PPID, Participation decision, Allocated trial, DATETIME
Potential participant completes ICF	PPID, Participation decision, Allocated Trial, Process evaluation participation decision, DATETIME
Validation email sent	PPID, DATETIME
Participant confirms participation by clicking link in validation email	PPID, PID, DATETIME
Participant receives blank demographics/measures form	PID, Form name, DATETIME
Participant submits complete demographics/measures form	PID, Form name, Form items, DATETIME
Participant randomised	PID, Allocated Trial, Allocated group, DATETIME
Participant logs into NEON Intervention	PID, SID, Access device, DATETIME
Participant shown recovery narrative	PID, SID, NID, RID, Route to Access, Categories, DATETIME
Participant provides narrative feedback	RID, Narrative feedback, DATETIME
Participant logs out of NEON Intervention	SID, DATETIME
Participant sent reminder about the NEON Intervention	PID, Reminder Communication Mechanism, DATETIME
Participant changes information on About Me page	PID, About Me, DATETIME
Participant requests payment of a voucher	PID, DATETIME
Participant selects button in footer	PID, Button name, RID (null if narrative not being viewed), DATETIME
Participant navigates to external URL	PID, URL, DATETIME
Participant confirms withdrawal request	PID, DATETIME
Participant blocks narrative	PID, NID, DATETIME
Participant bookmarks narrative	PID, NID, DATETIME
Participant unblocks narratives	PID, DATETIME
Non-participant safety event reporting form submitted	Safety event type, Caused by study, Date of event, Location of event, All other text on form, DATETIME
Participant safety event reporting form submitted	PID, Safety event type, Caused by study, Date of event, Location of event, All other text on form DATETIME

The following terms describe information logged through this process:

- DATETIME: Date and time that an event took place. Minimal recorded accuracy of one second (1 s)
- PPID: Potential participant ID. Temporary ID allocated to potential participant so as to link data they provide into trial records. Not linked to any identifiable data until consent is given
- How Found: Primary care. Secondary care. Online advert [name provider], Other
- Research site: Null, [any of the current secondary care research sites]
- Allocated trial: NEON Trial, NEON-C Trial, NEON-O Trial, Ineligible
- PIS item number: Number of the item being read on the PIS (“1”, “2”, etc.)
- PIS action: Scroll up, Scroll down
- PIS item action: Expand, Collapse
- Participation decision: Participate, reject
- Process evaluation participation decision: Yes, no
- PID (Participant IDentifier): The unique ID allocated to each participant after informed consent has been given
- Form name: Demographics, MANSA, CORE-10, Herth, MHCS, MIS, EQ-5D, CSRI
- Form items: All items on a demographics or measures form
- NID (Narrative IDentifier): The unique ID allocated to each recovery narrative by the NEON study team before uploading to the NEON Intervention
- RID (Request IDentifier): The unique ID allocated to each recovery narrative request
- SID (Session IDentifier): The unique ID allocated to each session of usage of the NEON Intervention
- Allocated group: Intervention, control
- Route to Access: Content-based match, Collaborative match, Random, Category, Hopeful, Bookmarked, First
- Access device: Mobile device, Computer
- Narrative feedback: As defined in the description of the intervention
- Reminder Communication Mechanism: Email, SMS, Facebook, etc.
- About Me: A vector of (name,value) pairs representing the set of values entered by the participant using the About Me page
- Button name: About Me, I’m upset, Welcome, Get me out of here
- Safety event type: Death, Life threatening event, Hospital admission, Hospital stay extension, Disability or incapacitation, Something else
- Caused by study: Yes, Unsure, No
- Date of event: Date that the safety event occurred (but not time)
- Categories: Vector of categories used to narrow down narrative, if category view used to find narrative
- Q5 responses: All responses provided to Q5 in the eligibility testing questionnaire.

Logs providing information about operation of the online system as a whole and of the matching algorithm will also be collected. These are summarised in Table 5.

**Table 5:** Logs of system operation

Item	Information logged
Heartbeat	DATETIME
Planned system close-down	DATETIME
Planned system restart	DATETIME
Unplanned system restart	DATETIME
Model retrained	DATETIME, Parameters
Narrative batch added	DATETIME, Narratives

The following terms describe information logged through these processes:

- Parameter list: A vector of <name,value> pairs describing the model produced by retraining
- Narratives: A list of NIDs added to the NEON Intervention.

The “heartbeat” provides a mechanism for understanding whether the online interface was available for participant use at any given time. It will be recorded at a minimum interval of 1 min (more regularly if possible within the technical constraints of the web server hosting the interface). This will be augmented by timestamps of the moment when the system restarts for any reason, to 1-s accuracy. Because crashes are caused by an unanticipated system failure, it is not technically feasible to record the precise moment when an *unplanned* system close-down occurred. If a *planned* system close-down takes place (e.g. for technical maintenance work), then this will be logged to 1-s accuracy.

#### Addition of new narratives to the NEON Intervention

New narratives may be added to the NEON Intervention as the trials proceed. This will allow for the optimisation of the NEON Intervention. The addition of new narratives would be important if interim analyses of demographic data show that participants have joined the trials from groups who are under-represented in the NEON Collection. It would also be important if some participants used the NEON Intervention so regularly that they were at risk of “running out” of new narratives to access. The addition of a batch of new narratives may incentivise re-engagement with the NEON Intervention, and prompts might be sent to draw attention to the presence of new narratives. If new narratives are added, they will be added in a small number of batches, at intervals of no less than 2 months. The date and contents of each batch will be logged in the SAP.

#### Operation of the matching algorithm

The NEON Intervention is a hybrid recommender system [54], which uses both collaborative filtering and content-based filtering to match narratives to users. When the user requests a new match, the list of narratives presented to the user will include a small number of items generated through each route. When narratives in the match are accessed, logging will incorporate an item indicating whether accessed narratives were recommended through collaborative filtering or content-based filtering, so that evaluation work can generate knowledge on their relative importance.

Recommendations based on collaborative filtering will identify narratives with characteristics which have previously received positive narrative feedback scores from users considered similar to the requesting user, using a distance metric [105] calculated from user characteristics listed in the personal profile and from narrative feedback scores. Recommendations based on content-based filtering will identify narratives whose content is predicted to appeal to the requesting user. This prediction will utilise a model developed through supervised machine learning [57].

The supervised training process for this model requires a training dataset, and a larger training set typically leads to a more effective model. All three trials will start with a single model trained from data collected during the intervention development phase of the NEON study (Slade, Rennick-Egglestone, Llewellyn-Beardsley et al: Using recorded mental health recovery narratives as a resource for others: Narrative Experiences Online (NEON) intervention development, submitted). This means that each trial will start with an identically parameterised model. This model will then be retrained using narrative feedback data provided by participants in each of the three trials. The models used in each trial will diverge, which is appropriate given the differing study populations. Retraining will take place regularly during the trial to maximise the benefits provided by retraining. Retraining will be conducted at least once during the internal pilot to assess the technical feasibility of retraining the algorithm whilst the NEON Intervention is still in use. At each retraining point, and in line with objective 6, all parameters defining the model will be logged, to allow for an understanding of how it has developed. The retraining protocol used during the trial will be logged in the SAP and included in the trial report.

The NEON Intervention is intended to support positive psychological change in the user. In order to enable any change to influence the matching process, at key points the user may be prompted to update user characteristics stored in his/her personal profile. This is most likely to lead to changes in the “Recovery status” or “Diagnosis” item. Prompts might occur immediately after filling out follow-up questionnaires at the 1 week and 12 weeksfollow-ups. They would be sent by any communication mechanism in the personal profile after receiving each batch of 25 narratives. Older narrative feedback scores will be reduced in weight and eventually discarded for the purpose of calculating preference similarity with other users in collaborative filtering. This reflects the assumption that psychological change might be manifested in changes in narrative feedback behaviour.

We anticipate that the effectiveness of the matching algorithm will improve over time. Recommendations made by collaborative filtering are likely to improve as new participants join the trials, since a larger number of participants may mean a more precise identification of others who are similar to the participant requesting a match. Recommendations made by content-based filtering are likely to improve as new content is added, as this provides for a greater pool of content, and as the model used to match participants to content is refined.

#### Qualitative process evaluation data collection

Qualitative process evaluation data will be collected from the following groups:

1. The first 30 intervention group participants in the NEON Trial will form an *internal pilot group*. Each will be offered the opportunity to take part in the process evaluation of the internal pilot, at least 2 weeks after randomisation.
2. Any NEON Trial participants who withdraw their consent for participation (the *withdrawal of consent group*) will be given the opportunity to tell the research team by email about how NEON can be improved. As they are no longer participating in the trial, they will not be interviewed but simply given the opportunity to provide feedback, which will be retained anonymously. Beyond clarifying any ambiguities, dialogue will not be entered into following the receipt of any feedback, other than to thank the persons for their feedback.
3. *Intervention group* participants for the NEON Trial will be offered the opportunity to take part in the process evaluation of the trial at the end of their 1-year participation. Interviewing will continue with this group until either theoretical saturation occurs or 50 interviews have been completed, whichever is sooner. Up to 20 participants in the NEON-C and NEON-O trials will be invited to a process evaluation interview.
4. If attrition is significant from the NEON Trial, interviews with up to 20 intervention group participants who only minimally used the intervention (*low engagement group*) will also be conducted, to understand the perspective of people who may not have found the intervention useful.

Participants in the *internal pilot*, *intervention* and *low engagement groups* will be contacted using their current contact information and asked to participate in an interview with a researcher by phone or secure video conference. At the start of the interview, the researcher will remind the participant that his/her participation is voluntary, that participation in the interview will be confidential and that only anonymised transcripts will be used in analysis. The participant will be asked to confirm that he/she consents to take part. These conversations will be captured in the audio recording of the interview. They will also be recorded onto a paper consent form by the researcher (Additional file 11). In all cases, participants can ask for an interview with a peer researcher if preferred. £20 will be offered to all participants as compensation for time and effort incurred, payable by electronic voucher or submission of a claim forms.

Interviews with the internal pilot group will focus on assessing the fidelity and safety of the intervention. This will allow the Programme Steering Committee (PSC) to make any necessary decisions about changes to study procedures following on from the internal pilot. The topic guide for internal pilot interviews will include questions intended to identify safety issues, to ascertain whether the NEON Intervention has been experienced as intended and to identify factors limiting fidelity, such as technical problems experienced by participants, or features of the interface limiting accessibility. Internal pilot interviews will take a maximum of 1 h.

Interviews with the intervention and low engagement groups will be broader for NEON Trial participants. A draft topic guide is shown in Additional file 12. In line with qualitative methodologies, the interview schedule will be refined over time. During the interview, some intervention group participants will be shown visualisations of logging data collected by the NEON Intervention and asked to explain interesting or unusual patterns, such as periods of very heavy or very light usage. This is a standard approach to enabling reflection on computer system usage [106], and it provides a mechanism for augmenting system logs with the cause of such phenomena. Interviews with the intervention group for the NEON-O Trial and NEON-C Trial will be shorter, and will focus on the acceptability of the intervention to these participants.

#### Resilience to unplanned system downtime

Occasional unplanned system downtime is to be expected with online interventions. A 2018 study of 32 web-hosting providers estimated 35 h downtime per year [107]. This makes it likely that some participants or potential participants will experience unexpected downtime whilst using the NEON Intervention.

Unexpected downtime might occur before consent has been provided by a potential participant. In this scenario, potential participants will need to complete eligibility checking processes again. Since no identifying information about potential participants can be collected until consent has been provided, it will not be possible to inform potential participants of the need to recomplete eligibility checking processes.

Unexpected downtime might occur after consent has been provided but before randomisation has been completed, e.g. partway through completing demographics and measures forms. In this scenario, a participant can continue completing demographics and measures forms the next time he/she logs in. Data will be retained from any completed forms and will not need to be re-entered. In the event of this scenario, the participant will be sent a reminder of the need to complete demographics and measures, by any contact mechanism they have specified on the consent form. This reminder will be sent at least 1 day after the first form was completed.

Unexpected downtime might occur after a participant has been randomised. This is inconsequential for trial processes, and hence no specific response is needed.

#### Safety event monitoring, response and reporting

##### Study principles

For all three trials, only serious adverse events (SAEs) will be monitored. This study policy has been agreed on with the PSC and study sponsor. It is consistent with HRA guidance, which indicates that only SAEs which are unexpected and related to the study should be reported to the Research Ethics Committee (REC) [108]. It has been informed by the online nature of the trials described in this protocol. Since the trials have no planned routine face-to-face contact with clinical or research staff, this would make routine monitoring for non-serious adverse events (AEs) intrusive. No event monitoring or reporting will take place after the trial has closed, even if a reported event pre-dated the end of the trial.

The HRA defines an SAE [108] as an untoward occurrence that:

1. Results in death
2. Is life threatening
3. Requires hospitalisation or prolongation of existing hospitalisation
4. Results in persistent or significant disability or incapacity
5. Consists of a congenital anomaly or birth defect; or
6. Is otherwise considered medically significant by the investigator.

The following AEs may result in an SAE and can be reasonably expected from this study, having been identified from the trial change model presented in Fig. 1. As they are expected, they will not be reported to the REC, in keeping with protocols established for other online studies [109]. All items in this list are specifically identified in the PIS as a possible harm of taking part in the trial so that potential participants can make an informed choice about participation:

- Feeling disconnected from others
- Feeling more pessimistic
- Feeling emotionally burdened
- Feeling inadequate
- Experiencing the release of uncomfortable emotions
- Engaging in harmful behaviours encountered in narratives.

In the event of uncertainty about whether an SAE should be categorised as unexpected, the CI can ask for a categorisation recommendation from the PSC chair.

All SAEs, regardless of categorisation, will also be reported in an anonymous form to the PSC in case a specific response is indicated, such as a change to trial procedures. If a Data Monitoring Committee (DMC) is formed, then the event will be communicated to the DMC with all participant details intact.

##### Safety event monitoring procedures

Since these are online trials with no face-to-face contact, it is likely that the CI will not become aware of all SAEs. The following procedures have been selected to create the maximum opportunity for SAE reporting:

- The version of the CSRI used in all three trials includes questions on nights spent in hospital and admissions to other hospital-based services. When used in the 12 months follow-up assessment, the retrospective period for the abridged CSRI is 12 months, which covers the entire duration of participation in the trial. As such, if a number other than 0 is entered for any of these questions, this could indicate that an SAE of type 3 as listed above has occurred, although it is still possible that the hospitalisation was planned, such as an operation for a physical health problem. Follow-up questions will be asked through the online form to collect sufficient details to allow for an event to be identified and categorised. Specific questions are included in Additional file 2.
- The splash page for the NEON Intervention will include a link to an online safety reporting form for use by participants or non-participants, which will allow SAEs of all types to be reported. Questions included in the safety reporting form are included in Additional file 13. After completing the form, the respondent will be given a message informing them that the report has been received. The respondent cannot be informed of how it will be processed, as this would breach the confidentiality of any participant that they reported on.
- The “About Me” page will include a link to an online safety reporting form, which allows participants to report SAEs (see Additional file 13). After completing the form, the respondent will be given a message thanking them for the report. Since this form is only accessible to the participant, it will exclude type 1 SAEs, as the participant cannot complete this form if he/she has died.

When an SAE is identified through an online form, an email notification of the SAE and all accompanying information will be sent to the CI and study coordinator.

Members of the study team or research site teams may also become aware of AEs through a direct communication from a participant or third party such as a family member or clinician. Some of these events may be classifiable as SAEs. Team members may become aware of these events:

- By email or phone, using contact details provided on the PIS or which are otherwise available publicly
- Through discussions in process evaluation interviews.

Any generic email address or phone number used by the study team will include a message indicating how regularly it will be monitored, and indicating that a response will be generated by the study team within a maximum of 3 days.

Since the study policy is only to monitor SAEs, the person receiving a communication will first assess the event. All individuals who might receive AEs will undertake Good Clinical Practice (GCP) training in advance of trial start to support them in understanding how to respond and categorise them, and appropriate supervision arrangements will be in place to support their practice and safety.

If the received communication relates to participant difficulties that do not reach the threshold for an SAE, the person sending the communication will be sign-posted to the support options identified through the “I’m upset” button, which is available to both the intervention group and the control group. If participant difficulties do reach the threshold for an SAE, the communicator will be asked about whether the study contributed to the event and also for further details of the event and its cause. The CI and study coordinator will be immediately informed of the event and the details that have been collected. If the person receiving the communication has any doubt about how to categorise the event, he/she will proceed on the assumption that it is an SAE, and the event will be referred to the CI.

In all reporting routes, if further information is needed about the event, the CI or a delegate will attempt to contact the respondent for further details. If contact cannot be established with the respondent within 3 days, the CI will log this and will make decisions based on available information.

##### Safety event categorisation and reporting

The CI will assess all events reported to him, using all available information. If the event is categorised as being an SAE that is both unexpected and related to the study, it will be reported to the REC, study sponsor, PSC and DMC if formed, within 15 days of the CI becoming aware of it. All SAEs will also be reported to the PSC chair, who will decide how to respond in collaboration with the CI. Options include no response; a direct recommendation to the CI from the PSC chair; or referral of the SAE to the full PSC or DMC for discussion and recommendation.

##### Emergency unblinding

The online interface used by the NEON trials will provide a function to reveal the allocation status of a single participant based on their ID, so as to enable emergency unblinding. This will only be accessible to the CI and study coordinator. Use of this function will be logged for the purposes of audit.

#### Data handling and record keeping

##### Data management

Electronic trial data will initially be collected through an application running on a secure web server and placing data into a secure database. The application, web server and database will be controlled by DRT Software, an IT supplier with a contractual arrangement to the study sponsor to control the data processing operations that can be performed, which will be specified in the delegation of duties log. The web server and database will be hosted by a web-hosting provider contracted to DRT Software. The web-hosting provider will be accredited an information security standard agreed on with the study sponsor in advance of trial start (such as Cyber Essentials or ISO 27001). The application will not be opened to trial participation until the study sponsor has agreed in writing that hosting arrangements meet appropriate levels of security for hosting sensitive personal information.

In the application, information that might identify an individual (e.g. email or phone numbers provided through the consent form) will be stored in a separate database from anonymous research data, linked only by ID. All information will be encrypted at rest using 256-bit Advanced Encryption Standard (AES) encryption or equivalent, or stronger. A backup process will be specified in a data management plan agreed on with the Pragmatic Clinical Trials Unit (PCTU), IT supplier and study sponsor, and all backups will be encrypted at rest. All information will be encrypted in transmission to and from the server using Hypertext Transfer Protocol Secure (HTTPS). At close of trial, all data stored on the web server, database and associated backups will be deleted using a thorough deletion protocol. When logging data from the trials is copied off the server, this will be logged so as to reveal who has accessed unblinded research data. For analysis and storage until end of the NEON study, anonymous research data will be transferred over HTTPS to a research server managed by the University of Nottingham.

The exception to these arrangements will be qualitative process evaluation interview data. Interviews will be carried out by either telephone or secure video conference. Recordings of interviews will be made on computers with encrypted hard drives and/or to encrypted audio recorders. As soon as possible, data will be copied onto research servers managed by the University of Nottingham and then deleted from the source device.

All data placed onto University of Nottingham research servers is backed up to two geographically redundant backup systems. Initially, the CI will only delegate access to the research team and study sponsor (the latter for the purposes of audit only). The research team incorporates staff at the University of Nottingham, PCTU, King’s College London, University of Manchester and Queen Mary University of London. If researchers work at sites other than the University of Nottingham, they will be able to download anonymous research for analysis using an individual associate account and a secure file transfer protocol. Alternatively, external researchers will be able to access data and analysis software using a remote desktop application, working over a secure connection. Consent logs will also be transferred to the same research servers, but to a different location and with access initially limited to the CI, and then delegated if necessary using the delegation of duties log.

All of these procedures will be approved in writing by the study sponsor before the trial opens to participants, and approvals will be deposited in the TMF. The study sponsor will be the Data Controller for personal information, and the IT contractor, web-hosting provider and University of Nottingham will be data processors. Data processing operations will only be entered into when satisfactory contracts are in place to control data processing operations, such as collaboration agreements. All processing of personally identifiable data will be logged, in keeping with General Data Protection Regulation (GDPR) requirements.

Where research data is provided directly by participants, e.g. by completing electronic forms presenting items from demographics or outcome measures, the design of these forms will be reviewed by a PCTU-approved statistician before use, and they will not be used to collect data until signed off by the statistician.

##### Confidentiality

Procedures for separation of identifying and anonymous data are recorded as above. All participation will be confidential. Confidentially will only be breached if, following a contact with a research team member, the CI decides that a participant is at risk to self or other, or has committed a notifiable offense. In this circumstance, consent logs will be searched for information that can be used to contact the person, and if possible the confidentiality breach will be agreed on with the participant in advance. If agreement cannot be reached, the CI may choose to pass on contact information to a relevant statutory authority.

##### Record retention and archiving

The body with responsibility for archiving of records beyond the end of the NEON study is Nottinghamshire Healthcare NHS Foundation Trust. For non-CTIMP studies, the funder indicates that the sponsor stipulates retention and archiving policies [110] (see subpage on archiving). The sponsor indicates a minimum retention period of 5 years for all records generated by this study.

#### Annual reporting

The CI will also send Annual Progress Reports to the trial REC and the sponsor using the HRA template. This will occur within 30 days of the anniversary of receiving the REC “favourable opinion”.

#### Monitoring and auditing

The sponsor or delegate retains the right to audit any study, study site or central facility. In addition, any part of the study may be audited by the funders where applicable. Audits will include the contents of the TMF and Investigator Site File (ISF) (to ensure compliance with Standard Operating Procedures [SOPs]).

#### Study committees

All three trials will be overseen by an independent Programme Steering Committee (PSC), which will function as a Trial Steering Committee (TSC). This committee has previously been convened by the CI. The chair of the PSC is Professor Sonia Johnson (Professor of Social and Community Psychiatry, University College London). Other members are Dr Tom Barker (Oxford NHS Foundation Trust), Dr Stephen Bremner (Senior Lecturer in Medical Statistics, Brighton & Sussex Medical School), Terry Harper (independent Patient and Public Involvement [PPI] representative, current mental health service user) and Paul Stevens (Peer Support Worker, Worcestershire Health and Care NHS Trust). Any changes in PSC membership will be reported to the funder, study sponsor, PCTU and REC. Minutes of PSC meetings will be placed in the TMF and reported to the funder, study sponsor and PCTU.

The CI and the PSC will decide whether to form a DMC, whose charter will be to identify ethical or safety issues emerging during the NEON Trial. The CI will inform the funder, study sponsor, PCTU and REC of a decision to form a DMC and its membership, or will provide a justification if a DMC is not formed. The DMC is the only body to have access to unblinded outcome data.

At 2 months, the study team will review recruitment and retention for all three trials. If needed, and with advice from the PSC, the study team will implement low-burden contingency plans such as extending the recruitment strategy. Examples would include obtaining support from charities in advertising the opportunity to participate.

Once the analysis of the internal pilot is complete (at 4 months or slightly later), the PSC will be presented with information about the fidelity, safety and recruitment/ engagement performance of the internal pilot. Safety information will include:

- Anonymous summaries of all SAEs received, including details of which (if any) were classified as being both related to the study and unexpected
- Anonymous analyses of elements of interview transcripts relating to safety.

The PSC will consider this information and decide whether to recommend changes to study procedures; whether to invoke remedial measures on recruitment and engagement; and whether to take forward data collected through the internal pilot into the full trial. For pragmatic trials, the latter is known to be a subjective judgement [111].

The PSC will also decide whether to invoke specific remedial measures to recruitment and engagement. The criteria for invoking these measures are as follows: recruitment, at or near 147 participants recruited within the first 4 months (i.e. 75% of target); engagement, 90% of intervention group participants view one narrative within 48 h of randomisation.

Remedial action to take would be decided by the PSC and identified in collaboration with the NEON Trial Management Group (TMG), which is composed of representatives of the NEON study research team, LEAP and PCTU. Examples of possible remedial strategies for recruitment include reducing the follow-up length to allow longer for recruitment or widening recruitment beyond England. Examples of remedial strategies for engagement include the addition of face-to-face facilitation by either researchers or local clinical staff. The analysis plan for the NEON Trial will be updated accordingly, and any changes would be summarised in the NEON Trial report.

At 6 months the PSC may again formally review recruitment and engagement in order to decide whether the NEON Trial should continue. The stop/go rule for recruitment will be attainment of, or clear trend towards, 206 (70%) of target 294 participants in this period. The stop/go rule for engagement will be attainment of, or clear trend towards, 80% of participants view one narrative within 48 h of randomisation. There are no stop/go rules for the NEON-C or NEON-O trials, but if the NEON Trial is stopped, these two trials will be stopped as well.

A representative of the study funder will be invited to all PSC meetings. Changes to the study considered minor (in the judgement of the PSC chair) will be considered implementable immediately. Major changes will be referred to the funder for review before implementation.

#### Operating procedures for the trials

Unless otherwise stated, the SOPs of Queen Mary University of London PCTU will be used for these trials. These include procedures for notifying relevant parties of protocol updates.

### Analysis

The trial statisticians conducting statistical analysis work and the NEON CI will be blinded in relation to allocation status until the statistical analysis plan (SAP) is signed off, all follow-up data is collected and data cleaning has occurred. Blinding will be through the use of access control lists in the file store used to aggregate data from the NEON Trials.

To maintain blinding for any interim reports, an independent statistician will prepare any information which requires knowledge of treatment allocations or involves data which would allow treatment allocations to be determined.

Statistical significance will be assessed at the 5% level, unless otherwise stated. Participant flow through the trial will be summarised in a Consolidated Standards of Reporting Trials (CONSORT) flow diagram [112].

#### Internal pilot evaluation for the NEON Trial

Analysis of the internal pilot of the NEON Trial will evaluate factors negatively affecting the fidelity of the NEON Intervention, identify any issues affecting the safety of participants and evaluate the recruitment process for the NEON Trial. These categories have been selected from the broader set of categories in the acceptance checklist for clinical effectiveness pilot trials (ACCEPT) [111].

There are three sources of data for the internal pilot evaluation: (1) anonymous transcripts of interviews with up to 30 intervention group participants, (2) system operation logs for the first 3 months of operation of the NEON Intervention, (3) logs of the first 3 months of participant flow through the NEON Intervention.

Factors affecting safety will be identified through an inductive thematic analysis of interview data. If analysis work identifies specific events which have occurred for individuals, then the procedure for handling these is described in the section on “Safety event monitoring procedures.”

Fidelity will be evaluated through (1) an inductive thematic analysis of interview data to identify factors negatively affecting fidelity and (2) a descriptive analysis of system operation logs to quantify system downtime and the impact on participants of downtime (e.g. by estimating numbers of participants using the system at each period where it became unavailable).

The recruitment process will be evaluated through the production of an interim CONSORT diagram [112] for the internal pilot and a table summarising recruitment routes used, and for each route identifying number of eligible participants, number of participants randomised, cost of recruitment.

#### Descriptive analysis for the NEON Trial

Only participants who have completed baseline measures and been randomised will be included in descriptive analyses.

Demographic and clinical outcomes at baseline will be summarised by treatment group. Demographic information will include all items collected on the baseline demographics form with age presented as mean (SD) and the remaining categorical variables presented as *n* (%). Clinical outcomes will include quality of life, as measured by the mean item score of the MANSA questionnaire; symptomatology, as measured by the total score of the CORE-10 questionnaire; hope, as measured by the total score of the Herth Hope Index; empowerment, as measured by the Mental Health Confidence Scale; and meaning in life, as measured by the mean item score on the *presence* and *search* subscales of the Meaning in Life Questionnaire. Higher scores indicate better outcomes in all but the CORE-10 measurement scales. Normally distributed data will be summarised by mean (SD); non-normally distributed data will be presented as median (interquartile range [IQR]).

For those in the intervention group, engagement will be summarised as the mean (and range) number of times a participant logs into NEON, receives a recovery narrative and provides a narrative feedback.

SAEs, withdrawals and timing of withdrawals from the trial will be summarised as *n* (%) by treatment group.

The number (percentage) of missing data on the demographics questionnaire and all clinical outcomes at baseline and follow-up timepoints will be summarised, and possible reasons for the missing data described and discussed.

#### Clinical outcomes evaluation

Only participants who have completed baseline measures and been randomised will be included in clinical outcomes analyses.

##### Primary outcome analysis for the NEON Trial

The primary outcome is the mean item score of MANSA at 52 weeks. Descriptive statistics of mean and median scores with SD and IQR will be presented by treatment group. Analysis will follow intention-to-treat (ITT) principles. The primary analysis will be a linear regression model of outcome at 52 weeks adjusting for baseline score and selected demographics. The results will be presented as adjusted difference in score at 1 year follow-up with associated 95% confidence intervals.

The missing data mechanism will be assumed to be missing at random (MAR) [113] unless evidence of violation is found. Under this assumption, missing data for the primary and key secondary analyses will be imputed using multiple imputation. The number of datasets generated will reflect the percentage of missing data present. Individual analyses on each imputed dataset will be combined using Rubin’s rules [113]. The imputation model will account for the longitudinal nature of the outcomes, and will include all variables contained in the substantive model and auxiliary variables which help to support the MAR assumption. Robustness of inferences to the MAR assumption will be explored through sensitivity analysis.

##### Secondary outcomes analysis for the NEON Trial

The secondary outcomes are total scores at 52 weeks of the CORE-10, Herth Hope Index and Mental Health Confidence Scale, and the mean item score on the presence and search subscales of the Meaning in Life Questionnaire. Descriptive statistics of mean and median score with SD and IQR will be presented by treatment group. Analysis will follow ITT principles and be conducted on the imputed dataset. The analysis of each outcome will be a linear regression model of outcome at 52 weeks adjusting for baseline score. The results will be presented as adjusted difference in score at 1 year follow-up with associated 95% confidence intervals.

The primary analysis will be repeated to include an interaction term between treatment and service user type at baseline (primary care service user, secondary care mental health service user, no prior mental health service use) to explore any differential treatment effect amongst service user types.

As a secondary analysis, to investigate differences in intervention effect over time, the primary outcome will be analysed by a linear mixed model with a random effect for participant.

Where outcomes are identified to be non-normally distributed, appropriate transformation or non-parametric analyses will be performed and detailed in the SAP.

##### Exploratory analysis of the NEON Trial

Exploratory analysis using the intervention group only will examine the patterns of intervention use, predictors of intervention use and association of usage with clinical outcome. Measures of usage include the number of stories accessed and the number of complete sets of narrative feedback stories provided.

Univariate associations between each usage measure and demographic and baseline clinical characteristics will be assessed by a Poisson regression model with the usage period (defined as the number of days between first and last log in) included as an offset term.

Linear or non-linear regression, as appropriate, will be used to assess the association of each usage measure with the MANSA score at 52 weeks.

To determine which variables and relationships may be important for consideration in the design and analysis of future trials, a model selection procedure will be conducted for the MANSA score at 52 weeks.

Since new narratives will be added to the NEON Intervention during the trial, and since retraining will be conducted on the machine learning algorithm used in the matching process, we will explore the possible impact on effect size of adding narratives and re-training the algorithm.

##### Exploratory analysis of the NEON-C and NEON-O trials

An exploratory analysis of outcome measures will be conducted using the same principles as for the NEON Trial. A preliminary effect size estimate will be made if recruitment and retention rates indicate this is statistically justified. This will support choice of outcome measures for a future definitive trial. All reporting will indicate that this was an exploratory trial.

#### Economic evaluation of the NEON Trial

Only participants who have completed baseline measures and been randomised will be included in economic analyses.

The economic evaluation will evaluate the costs of offering the NEON Intervention. It will present results with set-up costs both included (sensitivity analysis) and excluded (base case). Expected costs of offering the intervention include costs of (1) developing the software, (2) operating the online intervention, (3) periodically upgrading the software and updating narratives and (4) hosting the software on a server (including maintenance costs, server space, technical support and licensing arrangements). The impact of receiving the intervention on service costs will also be calculated, using service use data collected through the abridged Client Service Receipt Inventory (CSRI).

##### Outcomes

Quality-adjusted life years (QALYs) will be calculated by attaching available utility weights to the health states generated from the EQ-5D-5 L, using area under the curve methods with an assumption of a linear change between timepoints.

##### Incremental economic analysis

The economic evaluation has been designed using standard reporting criteria [114].The estimation of cost-effectiveness ratios will be carried out using the payer’s perspective (NHS England). Incremental cost-effectiveness ratios will be calculated in the event of the intervention having higher costs and better outcomes (based on EQ-5D-5 L and MANSA). Disaggregated costs and outcomes, and both deterministic and probabilistic incremental cost-effectiveness ratios, will be presented. The base case analysis will incorporate only NHS and Personal Social Services (PSS) costs and will express costs incurred in terms of QALY gain. Uncertainty will be addressed by generating cost-effectiveness planes from bootstrapped resamples. Cost-effectiveness acceptability curves will be constructed to show the probability that the intervention is cost-effective for different cost-per-QALY thresholds.

Further sensitivity analyses will examine (separately and together) the effects of expressing incremental changes in cost in terms of changes in MANSA; incorporating lost work; including intervention set-up costs; and variability in service delivery and uptake as determined during the RCT and process evaluation. Given the expected attrition of service user response, standard multiple imputation approaches to missingness will be employed and the effects on cost-effectiveness estimates will be examined.

#### Process evaluation analysis

Process evaluation analysis for the NEON Trial will follow best practice guidance [115, 116]. It will consider fidelity, reach, dose and adherence and also acceptability for the NEON Intervention. It will also evaluate the NEON change model for the study population and identify any safety issues relevant to future use of the NEON Intervention. The analysis will integrate quantitative data collected electronically through the NEON Intervention and qualitative data collected through interviews with three groups (withdrawal of consent, intervention, low engagers). Across all process evaluation analysis work, a particular focus will be on understanding the experience of participants who have never used mental health services, to generate knowledge about usage of the intervention by a large and under-researched group. This will amount to a planned subgroup analysis of all participants who self-identify as a member of this group through the trial demographics form. Process evaluation analyses for the NEON-O Trial and NEON-C Trial will be much more limited and will primarily focus on acceptability, through the analysis of qualitative interview data and logged intervention usage data.

##### Fidelity

We will evaluate factors that enabled or blocked usage of the NEON Intervention as a technology for delivering recorded recovery narratives. This will include quantitative analyses of logging data to identify periods of uptime and downtime for the NEON Intervention and the implications of participation for downtime, and the identification of factors enabling or blocking usage through interview analysis, such as difficulties obtaining access to computers or networks as a form of digital exclusion [117].

##### Reach

The reach of the NEON Trial will be investigated by analysing sociodemographic, clinical and geographic characteristics of participants to consider representativeness. For example, the sociodemographic profile of participants will be investigated to identify if there are groups who would be expected to but are not accessing the intervention, supplemented by qualitative data revealing any specific problems with accessibility.

##### Dose and adherence

There is no desired “dose” of recovery narratives received and no desired pattern of adherence to treatment, as even receiving just one recovery narrative might produce life-changing results. Rather, since no definitive trial of the use of recorded recovery narratives in the treatment of psychosis has been conducted, the aim of this part of the process evaluation will be to provide an understanding of patterns of receiving recovery narratives, and to explore their relationship to outcomes. This will support future clinical and non-clinical use of the NEON Intervention.

To provide an understanding of how the intervention was used, summary statistics will be presented for intervention use using data collected in the NEON Intervention logs. Duration of engagement will be calculated as number of days from baseline to final narrative received for each participant. Number of recovery narratives accessed per week of the intervention will be calculated using completion of all three narrative feedback questions as an indicator that the narrative has been processed in full. Summary statistics showing number of recovery narratives accessed in total versus number of recovery narratives accessed and feedback provided for will be presented.

#### Acceptability

The acceptability of the intervention will be evaluated through a deductive analysis of interview data. This will utilise existing frameworks [118] to structure transcript fragments.

##### Evaluation of the NEON change model

The NEON change model will be evaluated through qualitative analysis of interview data. Thematic analysis will be applied, using a hybrid deductive-inductive approach. An initial coding framework will be produced using concepts named in the trial change model, and this will be extended inductively by application to interview data, to allow accounting for unanticipated forms of change. Analysis will be supplemented by consideration of quantitative data, e.g. the extent to which high connection was associated with high hope-promotion, as assessed using narrative feedback scores.

##### Safety of the NEON Intervention

Factors relating to the safety of the NEON Intervention will be evaluated through qualitative analysis of interview data. Thematic analysis will be applied, using a hybrid deductive-inductive approach. This will begin with codes relating to safety developed through the qualitative analysis of the internal pilot (if any), which will be extended inductively through application to interview data.

#### Evaluation of the supervised machine learning algorithm

The performance of the selected supervised machine learning algorithm in training a model to do content-based recommendation will be evaluated, led by Jeroen Keppens, a machine learning expert who is an investigator on the NEON study. Firstly, models used by the algorithm during the NEON Trial will be inspected and described, providing information about types of matching data that were influential/not influential in the NEON Trial matching process. The generalisations generated by the model will be compared against first principles by examining whether they fit the domain literature and patterns observed by mental health researchers in this study.

The performance of the supervised machine learning algorithm will be evaluated by comparing its performance to that of a range of standard supervised machine learning algorithms suitable for recommendation systems. To enable this comparison and to ensure that the evaluation assesses how well each algorithm generalises to unseen data, all available recovery narrative feedback data generated during the NEON Trial will be partitioned into a training dataset and a validation dataset. This process is repeated over different partitions of the dataset into training and validation datasets [119].

The performance evaluation of each algorithm will utilise a vector metric calculated from logged narrative feedback data. Better algorithms will be defined as those that recommend narratives that score highly on one or more of these criteria. To assess the quality of a model produced by a machine learning algorithm, standard assessments of precision and recall in reproducing a top-N of narratives will be employed [120].

## Discussion

### Approaches to reducing bias

The following approaches to reducing bias in clinical trials [121] have been adopted for all three trials.

#### Selection bias

To minimise baseline differences between groups, all participants will be randomly allocated using an allocation algorithm validated by the overseeing Pragmatic Clinical Trials Unit (PCTU).

#### Detection bias

To eliminate any differences in how outcomes are measured, all participants will only provide outcome data through an online interface, which will be identical for each group.

#### Attrition bias

To reduce differences in attrition between the control and intervention groups, control group participants will be told that they will receive access to the NEON Intervention after the primary endpoint, and all participants will be compensated for the time and effort of providing outcome data.

#### Reporting bias

International Standard Randomised Controlled Trials Numbers (ISRCTNs) have been registered for all three trials; the trial report will be published open access; and a summary of trial results will be provided on the NEON study website.

#### Contamination bias

To avoid contamination through being allocated to the intervention group in one trial and the control group in another, individuals will only be allowed to take part in one trial.

Contamination is otherwise possible in three ways:

1. A participant allocated to the control group could repeatedly register until allocated to the intervention group. This will be minimised by not allowing the creation of a new login using the same email address as an existing login.
2. A participant allocated to the control group could request and use the login details from an intervention group participant. This risk is low because recruitment is online, so participants will not generally know who else is participating. This will be further minimised by asking all participants not to share their login details.
3. Deciding to participate in the trial might prompt participants to access publicly available narrative repositories (e.g. YouTube), or a participant may receive narratives through clinical care (e.g. from a peer support worker). To monitor this form of contamination, all participants will be asked at follow-up about access to recovery narratives outside of the NEON Intervention.

### Support for safe interaction with the NEON Intervention

A broad range of measures have been incorporated into the design of the NEON Intervention and the study procedures for the three trials to enable safe usage. These approaches have been developed in consultation with the NEON LEAP and NEON International Advisory Board (IAB).

1. Potential participants will be informed through the Participant Information Sheet (PIS) about forms of harm that can be caused by receiving recovery narratives, to enable an informed choice about participation. This information draws on the results of research conducted by the NEON study [4, 5]. It will provide participants with knowledge to support self-management.
2. All narratives used in the NEON Intervention will first be characterised by researchers, using the Inventory of the Characteristics of Recovery Stories (INCRESE) , which has been developed by the NEON team. This includes items to identify narratives which should have content warning(s) about potentially distressing content. For the NEON trials, the characteristics of each narrative included in the NEON Intervention will be rated by a single researcher using INCRESE. Additionally, since our testing indicates that it is not possible to identify narratives requiring content warning with 100% accuracy because of differences in rater interpretation about content, items relating to content warnings will be second-rated by a separate rater. If either rater identifies a content warning as relevant, it will be applied. Narratives including distressing content should not be excluded, as content which is distressing for some will provide benefits to others [5].
3. The Initial information page, which is the first page encountered in the NEON Intervention, will enable intervention group participants to specify categories of potentially distressing content that they wish to avoid, and this preference can be updated through the About Me page. Narratives identified by raters as including that content will never be visible to participants. Being able to exclude all narratives including a particular type of content is a form of self-management.
4. If a narrative is identified as including a type of potentially distressing content which has not been excluded by a participant, then before the participant accesses this narrative, he/she will be shown a content warning and given the choice of whether to continue or not.
5. Users can block a narrative that they find distressing, and this narrative will not be listed in the NEON Intervention until unblocked.
6. Self-management strategies identified by participants at first login are shown to them when they click the “I’m upset” button.
7. At first login, the Useful Information page will provide brief advice on how to handle difficult emotional responses to narratives, and further advice will be provided on the “I’m upset” page. This information will always be available through the footer of the NEON Intervention.
8. A button labelled “Get me out of here” is provided in case a participant feels distressed and wants to quickly close the interface, or if the interface is being accessed in a public setting and a participant does not want others to know about their usage.
9. Topic guides of interviews with the internal pilot, intervention and low-engagement groups will consider safety issues, and analysis of interviews allows the opportunity to refine the NEON Intervention or trial processes in light of unanticipated safety issues.

Our decision to use content warnings (also known as trigger warnings) has drawn on a review of the literature on content warnings, conducted through systematic searches of the MEDLINE, Cumulative Index to Nursing and Allied Health (CINAHL) and PsycINFO databases. These have identified four publications presenting empirical evidence on the impact of real-world usage of content warnings. These studies show that content warnings can reduce harm by reducing stress in students with post-traumatic stress disorder (PTSD) diagnoses [122] and by reducing negative emotions produced by engaging material [123]. They can be useful if provided well in advance of discussions in an educational context [124] and can reduce negative emotions and signal supportive environments, which may promote engagement with otherwise distressing material in the long run [123]. They can however also increase avoidance, which may prevent people from learning to cope with distressing content [123]. Only one RCT has been conducted examining the psychological effects of issuing content warnings [125]. This trial found that warnings produced a small increase in self-reported anxiety after reading potentially disturbing literary passages amongst people who believed words can cause harm. It also found a slightly increased perceived risk of suffering long-term emotional harm in the wake of a traumatic event.

### Safety of narrative donors

The NEON Intervention presents recovery narratives from the NEON Collection. A protocol for the management of the NEON Collection was previously approved by the UK Health Research Authority (HRA). The IRAS ID for this approval is 24734. The protocol was given favourable opinion by the West London & Gene Therapy Advisory Committee (GTAC) Research Ethics Committee. The REC reference is 18/LO/0991. This approved protocol supports the safety of individuals whose narratives are incorporated in the NEON Collection in the following ways:

- Narratives are only included in the NEON Collection where valid consent has been documented. Narratives sourced from public collections are only included if the narrator has provided his/her publisher with consent for public reuse, or if the narrator has been approached and provided explicit consent for use in the NEON Collection. Where a narrative is donated directly to the NEON Collection, informed consent is provided through an online consent form. Narrators can withdraw consent for inclusion whilst the NEON Collection is in use.
- Narratives are incorporated exactly as originally published.
- All candidate narratives are assessed by at least one researcher against inclusion and exclusion criteria published at http://researchintorecovery.com/neoncollection. If eligibility is unclear, a final decision is made by a Collection Steering Group consisting of four members of the LEAP and two researchers.
- Third parties can request withdrawal of a narrative, e.g. if a narrator has died, or if they assert that the narrator did not have capacity to consent to inclusion of their narrative. They do not have an automatic right to withdrawal, in order to protect the rights of the narrator to have their story told. A final decision on third-party requests is made by the Collection Steering Group.

These strategies were developed with advice from the LEAP. Three members of this panel have published their own recovery narratives.

## Trial status

The current version of the trial protocol is v4.0, dated 16 January 2020.

The trial opened to recruitment on 9 March 2020. Planned recruitment close is 30 April 2021. Planned trial end is 30 April 2022.

## Supplementary information

**Additional file 1. **Items in the NEON Intervention personal profile**.** Defines all items included in the personal profile for each NEON Intervention user.

**Additional file 2. **Demographics and measures forms**.** Defines all online demographics and measures forms used in the NEON Trials.

**Additional file 3.** Promotional principles. Defines principles to which all promotional messaging for the NEON trials must conform.

**Additional file 4.** Recruitment poster for all NEON trials. Integrated recruitment poster to promote all trials.

**Additional file 5.** Recruitment poster for the NEON Trial. Recruitment poster specific to the NEON Trial.

**Additional file 6.** Recruitment poster for the NEON-C Trial. Recruitment poster specific to the NEON-C Trial.

**Additional file 7.** Recruitment poster for the NEON-O Trial. Recruitment poster specific to the NEON-O Trial.

**Additional file 8.** Participant messaging in the NEON trials. Defines messages displayed to participants in the NEON trials.

**Additional file 9.** Online Participant Information Sheet. Text and layout for the online Participant Information Sheet used in the NEON trials.

**Additional file 10.** Online Informed Consent Form. Text and layout for the online Informed Consent Form used in the NEON trials.

**Additional file 11.** Process evaluation Informed Consent Form. Paper Informed Consent Form for use in process evaluation interviews.

**Additional file 12.** Process evaluation topic guide. Topic guide for process evaluation interviews.

**Additional file 13.** Safety event reporting forms. Defines questions used in two safety reporting forms.

## Data Availability

Anonymous and pseudonymous elements of the datasets used and/or analysed during the study will be available on request from the study team before the NEON study ends, although requests may be refused whilst research publications are being generated. After the NEON study ends, anonymous and pseudonymous research data will be available from the study sponsor on reasonable request until the end of the retention period, but request may be refused if NEON study investigators are still generating research publications from this data. After the retention period, availability through the study sponsor or Chief Investigator may be provided at their discretion. Contact the study sponsor through Research@nottshc.nhs.uk.

## Abbreviations

ACCEPT: Acceptance checklist for clinical effectiveness pilot trials
AE: Adverse event
AIC: Akaike information criterion
CSRI: Client Service Receipt Inventory
CTIMP: Clinical Trial of an Investigational Medicinal Product
DHT: Digital healthcare technology
DMC: Data Monitoring Committee
GCP: Good Clinical Practice
GP: General practitioner
HCI: Human-computer interaction
HRA: Health Research Authority
HTTPS: Hypertext Transfer Protocol Secure
IAB: International Advisory Board
IAPT: Improving Access to Psychological Therapies
ICF: Informed Consent Form
INCRESE: Inventory of the Characteristics of Recovery Stories
IRAS: Integrated Research Application System
ISRCTN: International Standard Randomised Controlled Trials Number
ITT: Intention to treat
LCRN: Local Clinical Research Network
LEAP: Lived Experience Advisory Panel
MANSA: Manchester Short Assessment of Quality of Life
MHRA: Medicines and Healthcare products Regulatory Agency
NEON: Narrative Experiences Online
NHS: National Health Service
NID: Narrative IDentifier
NIHR: National Institute for Health Research
PCTU: Pragmatic Clinical Trials Unit
PIC: Participant Identification Centre
PID: Participant IDentifier
PIS: Participant Information Sheet
PSC: Programme Steering Committee
PSS: Personal Social Services
REC: Research Ethics Committee
SAE: Serious adverse event
SAP: Statistical analysis plan
SUSAR: Suspected unexpected serious adverse reaction
TAG: Threshold Assessment Grid
TMF: Trial Master File
TMG: Trial Management Group
TSC: Trial Steering Committee
