# Supplementary material for: Impact of receiving recorded mental health recovery narratives on quality of life in people experiencing psychosis, people experiencing other mental health problems and for informal carers: Narrative Experiences Online (NEON) study protocol for three randomised controlled trials
Source: Trials. 2020 Jul 20;21:661. doi: 10.1186/s13063-020-04428-6 (PMC7370499; doi:10.1186/s13063-020-04428-6)
Supplement: Supplementary file 1 — Additional file 1. Items in the NEON Intervention personal profile. Defines all items included in the personal profile for each NEON Intervention user. [file 13063_2020_4428_MOESM1_ESM.pdf]

Contents of the NEON Intervention personal profile

The personal profile used by the NEON Intervention stores the following items.

User characteristics [directly editable]

Matching data. None mandatory, and defaults to “Prefer not to say”.

Gender. Prefer not to say; Female; Male; Other

Age. Prefer not to say; 18-25; 26-40; 41-65; 66+

Ethnicity. Prefer not to say; Asian; Black/African/Caribbean; Dual/multiple ethnic group; Other ethnic group; White

Sexuality. Prefer not to say; Bisexual; Gay man; Heterosexual; Lesbian/gay woman; Other

Stage of recovery. Prefer not to say; Not yet thinking about recovery; Thinking about recovery; Working on recovery; Rejects recovery

Disability. Prefer not to say; None; Visual difficulties; Hearing difficulties; Mobility/ stamina difficulties; Cognitive difficulties; Self-care difficulties [multi-select]

Diagnosis. Prefer not to say; None; Neurodevelopmental disorder; Eating or food-related disorder; Mood disorder; Personality disorder; Obsessive-compulsive disorder; Schizophrenia or other psychosis; Trauma/stress disorder; Substance- disorder; Rejects diagnostic framework [multi-select]

Narrative format preference: Prefer not to say; Narratives containing text; Narratives containing audio; Narratives containing moving images; Narratives containing static images [multi-select]

Narrative content to block [directly editable]

Not mandatory. Defaults to empty list. The participant is allowed to block narratives containing this content for safety reasons.

Abuse or sexual violence
Loss of life or endangerment to lifeSelf-harm including eating disorders
Violence or aggression
Injustice, prejudice and discrimination

Narrative formats to block [directly editable]

Not mandatory. Defaults to empty list. The participant is allowed to block narratives of these formats since they might have disabilities that disrupt processing of media (e.g. dyslexia), or since they may have to access narratives in settings that preclude some formats (e.g. if they are always accessing a narrative in a public library, and hence cannot receive narratives incorporating audio or video).

Narratives containing:

Text
Audio
Moving images
Static images
[multi-select]

Strategies to be reminded about in case of distress [directly editable]

Not matching data. Not mandatory.

Free text.

Personal contact information [directly editable]

Login email address. Populated from the consent form and fixed. [mandatory]

Mobile telephone number. Populated from the consent form.

Facebook account.

Twitter account.

Stored in personal profile but not directly editable

List of favourite narrative

List of blocked narratives
