# Supplementary material for: Impact of receiving recorded mental health recovery narratives on quality of life in people experiencing psychosis, people experiencing other mental health problems and for informal carers: Narrative Experiences Online (NEON) study protocol for three randomised controlled trials
Source: Trials. 2020 Jul 20;21:661. doi: 10.1186/s13063-020-04428-6 (PMC7370499; doi:10.1186/s13063-020-04428-6)
Supplement: Supplementary file 3 — Additional file 3. Promotional principles. Defines principles to which all promotional messaging for the NEON trials must conform. [file 13063_2020_4428_MOESM3_ESM.pdf]

Principles of promotional material

Principle 1: If the communication mechanisms affords it (e.g. on a poster), then promotional material will include the study sponsor logo and name, the study logo, and details of the approvals received by the study (e.g. Health Research Authority, name of REC offering favourable opinion, study sponsor)

Principle 2: If the communication mechanism does not afford it (e.g. in a tweet with limited characters), then the promotional material will always include a link to a page that provides the same information as in principle 1.

Principle 3: Promotional material will clearly indicate that we are looking for participants for a clinical trial (“trial” may be used as an informal synonym of “clinical trial”).

Principle 4: Promotional material will clearly indicate that the trial involves receiving recovery stories (the term “recovery story” has been selected as a more accessible synonym than “recovery narrative”).

Principle 5: If images of people are included in the promotional material, then these will only be included if appropriate documented consent is in place for this usage, e.g. if the image was specifically captured for inclusion in the promotional material, or if it was licensed from an image library (e.g. a stock image of two people working on a computer)

Principle 6: If the communication mechanism affords it (e.g. a poster), then typography and layout will be selected to be dyslexia-friendly and appropriate for people with red-green colour blindness, as this is the most common form of colour-blindness

Principle 7: Promotional material will not be placed by the study team into settings where people have a reasonable expectation of privacy (such as Facebook groups closed to public membership). Promotional material may be placed into private settings only by people who have a reasonable, pre-existing right of access to those settings (such as existing members of Facebook groups)

Principle 8: Promotional material will not be made available in languages other than English, even on request, as fluency in English is an inclusion criteria for all three trials.

Principle 9: All promotional material used by the study will be archived in the TMF, and hence will be open to audit by the study sponsor, so that the study sponsor can confirm that these principles have been applied.
