# Supplementary material for: Impact of receiving recorded mental health recovery narratives on quality of life in people experiencing psychosis, people experiencing other mental health problems and for informal carers: Narrative Experiences Online (NEON) study protocol for three randomised controlled trials
Source: Trials. 2020 Jul 20;21:661. doi: 10.1186/s13063-020-04428-6 (PMC7370499; doi:10.1186/s13063-020-04428-6)
Supplement: Supplementary file 6 — Additional file 6. Recruitment poster for the NEON-C Trial. Recruitment poster specific to the NEON-C Trial. [file 13063_2020_4428_MOESM6_ESM.pdf]

Do you care for someone with mental health problems?

NEON
Narrative Experiences Online

COULD REAL-LIFE RECOVERY STORIES HELP YOU?

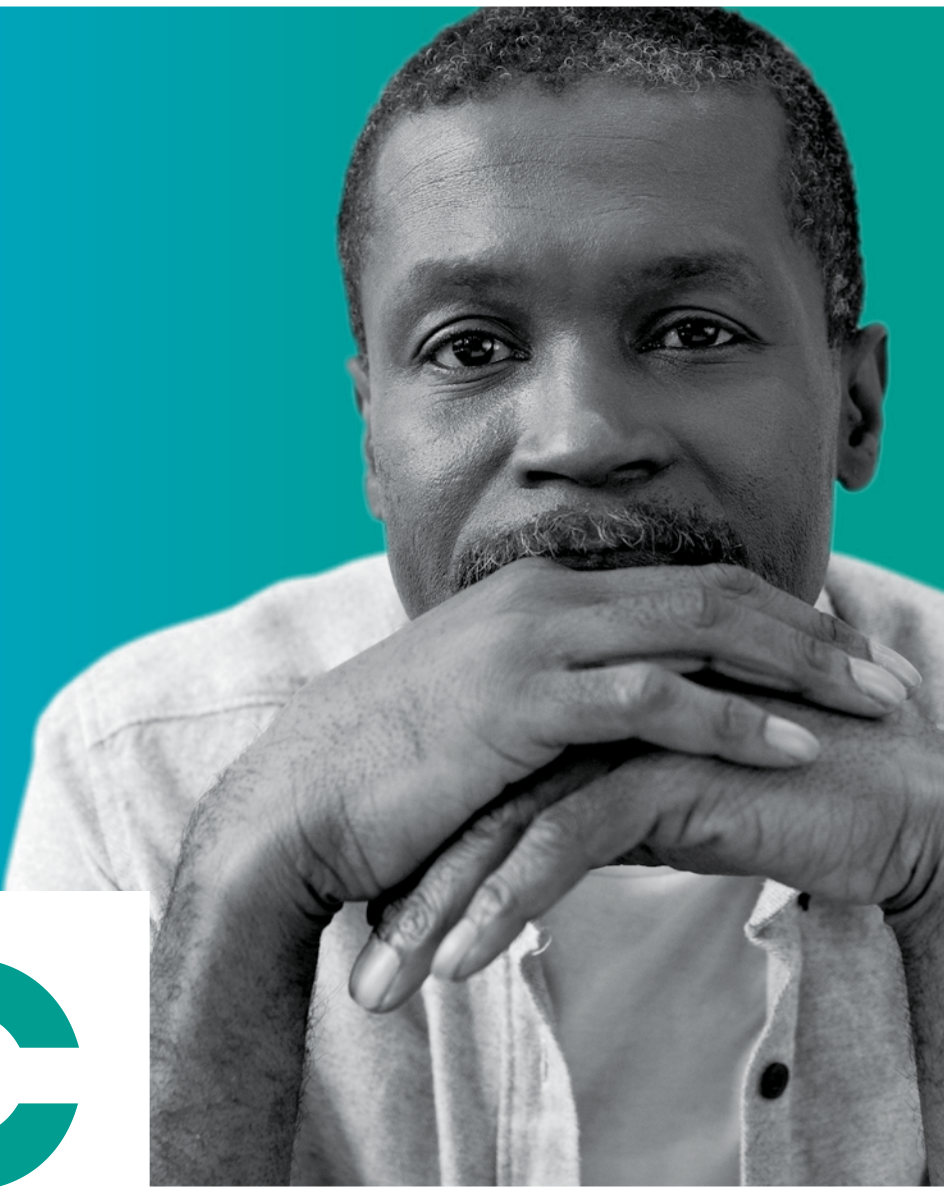

Join the NEON-C Trial

Find out more at **recoverystories.uk**

NEON is funded by the NIHR (RP-PG-0615-20016).
This study has been given favourable opinion by Leicester Central REC, and has been approved by the HRA. The study sponsor is Nottinghamshire Healthcare NHS Foundation Trust.
File: NEON-C Trial. Poster MEM. v2 20.12.19.

NHS
Nottinghamshire Healthcare
NHS Foundation Trust
