# Supplementary material for: Impact of receiving recorded mental health recovery narratives on quality of life in people experiencing psychosis, people experiencing other mental health problems and for informal carers: Narrative Experiences Online (NEON) study protocol for three randomised controlled trials
Source: Trials. 2020 Jul 20;21:661. doi: 10.1186/s13063-020-04428-6 (PMC7370499; doi:10.1186/s13063-020-04428-6)
Supplement: Supplementary file 8 — Additional file 8. Participant messaging in the NEON trials. Defines messages displayed to participants in the NEON trials. [file 13063_2020_4428_MOESM8_ESM.pdf]

Participant messaging text

Message 1

The following questions will allow us to decide whether you are eligible to take part in any of the clinical trials being conducted by the NEON study. Please only complete them if you are interested in taking part in a clinical trial. If you don't want to take part in a clinical trial, but would like to learn more about the NEON study, then please visit the study website <link to http://researchintorecovery.com/neon>.

Message 2

Thank you for answering our questions. Sadly, you are not eligible to take part in any of the NEON trials, and you should only complete the questions again if your circumstances have changed. If you would like to see some recovery stories, you might take a look at a list we have created: https://www.researchintorecovery.com/public-recovery-stories.

Message 3

Thank you for considering involvement. If you change your mind you are welcome to return and re-register. You can safely close this window.

Message 4

Great, you've now joined the trial. We would now like to collect some information about you to help us evaluate the NEON Intervention. This will take about half an hour to complete. It will not be used to match you to stories if you are given access to the NEON Intervention, and it cannot be used to identify you. Once you have completed all the information, you can claim a £20 voucher as thanks for the time and effort that it has taken you. Some questions might feel a little sensitive, so you might choose to do this in a private place.

We will contact you in 1 week, three months and one year to ask you to complete the online survey again. You can claim a further £20 each time you complete the online survey.

Message 5

Thank you for your responses. You have been randomly selected to receive access to the NEON Intervention after one year. Until then, you are still an important part of our trial, and we want you to continue taking part. We will contact you to ask you to complete our survey again in 1 week, 3 months and 1 year. You can still claim £20 after each survey. After one year you will receive access to the NEON Intervention for as long as it is available. You can safely close this window now.

Message 6

Thank you for your responses. You have been randomly selected to receive immediate access to the NEON Intervention, and please use it as little or as much as you want, and we will ask you to complete our survey again in 1 week, 3 months and 1 year.Please do not share your login details with anyone else.

Message 7

You have asked to withdraw from this study. If you confirm this decision then all data that might identify you will be deleted and only anonymised data will be kept. You will no longer have access to the NEON intervention.

If you are thinking about withdrawing and would like to discuss this with a NEON researcher please click here <button 1>

If you are sure you want to withdraw please confirm your decision by clicking here <button 2>

If you do not want to withdraw, please click here: <button 3>

Message 8

We're sorry to see you go. We have deleted all data that might identify you, including your login details. If you are leaving because of something to do with NEON and would like to tell us anything about how NEON could be improved, please <contact us>. We are particularly keen to hear if there is anything about NEON which has made you feel unsafe.

Message 9

Thank you. We will be in touch with you as soon as possible using the contact information listed in the About Me page. If needed, please update your preferences now so we have the correct contact information for you.

Message 10

Thank you for taking part in our trial. The NEON study is coming to an end, and so your access to the NEON Intervention will stop working on <date>. We've created a list of public recovery stories in case you want to look at more examples: http://www.researchintorecovery.com/public-recovery-stories

Message 11

Thank you for taking part in our trial. The NEON study has now concluded, and so your access to the NEON Intervention has ended. If you would like to look at more recovery stories, we have created a list: http://www.researchintorecovery.com/public-recovery-stories
