# Supplementary material for: Impact of receiving recorded mental health recovery narratives on quality of life in people experiencing psychosis, people experiencing other mental health problems and for informal carers: Narrative Experiences Online (NEON) study protocol for three randomised controlled trials
Source: Trials. 2020 Jul 20;21:661. doi: 10.1186/s13063-020-04428-6 (PMC7370499; doi:10.1186/s13063-020-04428-6)
Supplement: Supplementary file 11 — Additional file 11. Process evaluation Informed Consent Form. Paper Informed Consent Form for use in process evaluation interviews. [file 13063_2020_4428_MOESM11_ESM.pdf]

Informed Consent Form for process evaluation interviews
Version 1.0. 15th November 2019.

This Informed Consent Form should be completed by the researcher conducting the interviews, as a record of the verbal consent that was provided by the participant.

The researcher should confirm each of the statements by placing their initials in each of the boxes below, and should sign and date the Informed Consent Form.

Please **initial** each of the following boxes:

1. The participant was reminded that their interview would be audio-recorded [1]
2. The participant was reminded that their participation in the interview is voluntary [2]
3. The participant was reminded that their participation in the interview will be treated as confidential, and that analysis will only be of anonymised transcripts. [3]
4. The participant confirmed their consent to take part in the interview. [4]
5. The participant's verbal confirmation of consent was captured on the audio recording of the interview. [5]

Name of researcher conducting interview: ……………………………………………………….

Signature of researcher conducting interview: …………………………………………………...

Date of interview: ……………………………………………………………………………………
