# Supplementary material for: Impact of receiving recorded mental health recovery narratives on quality of life in people experiencing psychosis, people experiencing other mental health problems and for informal carers: Narrative Experiences Online (NEON) study protocol for three randomised controlled trials
Source: Trials. 2020 Jul 20;21:661. doi: 10.1186/s13063-020-04428-6 (PMC7370499; doi:10.1186/s13063-020-04428-6)
Supplement: Supplementary file 12 — Additional file 12. Process evaluation topic guide. Topic guide for process evaluation interviews. [file 13063_2020_4428_MOESM12_ESM.pdf]

Topic guide for process evaluation interviews

Interviews will include some of the following questions, depending on the interview group. The topic guide will evolve over time in the light of emerging findings from interim qualitative analysis. Interviewers will refine how questions are worded, according to the communications needs of participants.

Topics are shown as bullet points, with example follow-up and probing questions which will be used to elaborate topics of interest.

- Initial engagement

How did you learn about NEON?
When you first heard about it, what made you interested in taking part?

- Consent procedures

Did you have any concerns about taking part? How effectively did information provided in the interface address these concerns?
Do you feel you fully understood what you were consenting to?
We used a consent form which gave only short summaries with the chance to open a more detailed description. What are your thoughts on this approach?

- Technical issues

What types of device have you used to access NEON? Have you encountered any difficulties in using these devices to access it?
Did you ever believe that the NEON Intervention wasn't working properly?
How did you find navigating around NEON?
How could we make NEON easier to use?

- Using NEON

Where have you been when you have accessed NEON? [e.g. at home, travelling, public libraries etc]
When you first started using NEON, were there any features that you found difficult or confusing? Are there any features that you still find difficult or confusing?
How regularly do you use the NEON Intervention? Would you like to use it less or more?

- Patterns of usage

<Participants shown a visualisation of their usage logs> Can you describe the context behind any significant features, such as periods of heavy or little use?

- Mechanisms of change

Have you noticed any changes to your thoughts, feelings or behaviours as a result of the stories you have seen in NEON? Can you say how the story led to the change?
Have any stories been particularly helpful to you? Have any been harmful?
Have you looked at any recovery stories outside of NEON?
<Participant shown list of recovery narratives that they have accessed> Can you identify a narrative that was particularly impactful? What was it that that made an impact? What type of impact did it make?

- Reach

<If relevant> Can you describe the problems in using NEON? Did you actively decide to stop using NEON? Why?
What would have made it easier for you to use NEON more often?
Who do you think NEON is most helpful for?

Are there any groups of people you think NEON might be particularly helpful for? Or harmful for?

- Safety issues

Did you feel distressed at any point whilst using NEON? What caused you to be distressed? How did you manage your distress? Did you use the “I’m feeling upset” page? How well did it work? What could be added to the NEON Intervention to make it safer?

- Perceptions of the matching algorithm

How do you feel about the matches generated by NEON? Were they good or bad? Do you have any concerns about the matching process?
