# Supplementary material for: Impact of receiving recorded mental health recovery narratives on quality of life in people experiencing psychosis, people experiencing other mental health problems and for informal carers: Narrative Experiences Online (NEON) study protocol for three randomised controlled trials
Source: Trials. 2020 Jul 20;21:661. doi: 10.1186/s13063-020-04428-6 (PMC7370499; doi:10.1186/s13063-020-04428-6)
Supplement: Supplementary file 13 — Additional file 13. Safety event reporting forms. Defines questions used in two safety reporting forms. [file 13063_2020_4428_MOESM13_ESM.pdf]

Anonymous safety reporting form

Display location: This is linked to from the splash page for the three NEON trials. It can be used, anonymously, by anyone to report a safety event.

Title: An incident has occurred for someone taking part in a NEON trial.

Subtitle: This form can be used to report problems that have been experienced by people who are taking part in one of the three NEON Trials (NEON, NEON-O, NEON-C). We use this information to help us monitor the safety of NEON and of individual participants. Please tell us as much as you can, as this will help us in responding effectively. Any information you tell us will be logged, and reported to the NEON Chief Investigator and others.

Q1: What has happened? [Response options: The participant has died, The participant has experienced a life threatening event, The participant was admitted to hospital, The participant was kept in hospital longer than planned or expected, The participant has been disabled or incapacitated, The participant has experienced something else (opens text box labelled: Please describe)]

Q2: In your opinion was this caused by their participation in a NEON trial? [Response options: yes, unsure, no]

Q3: When did this occur? Please give us your best guess if you are not sure.
[Date entry field]

Q4: Where did this occur? [Multiline free text]

[If the response to Question 2 is "yes" or "unsure"] Q5: Please tell us how you think the trial might have caused this. [Multiline free text]

Q6: Who has this happened to? Please let us know any different names used by this person. [Multiline free text]

[Unless Q1 response is "The participant has died"] Question 7: How can we contact them? Please provide all contact details you know of for the person
[Multiline free text]

Q8: How is it best for us to contact you if we need more information? [Multiline free text]

Response on completion: Thank you for taking the time to complete this form. The information that you have provided has been immediately passed to the NEON Chief Investigator and study coordinator, and will be processed rapidly.

[Notes: Q1 and Q2 defaults to "Select an answer from the list", and a response option must be selected before the form can be submitted. Q3 defaults to a blank date, and a date must be entered before the form can be submitted. Q4-Q8 default to blank text if enabled, and some text must be entered before the form can be submitted.

Safety reporting form for participants in a NEON trial

Display location: This is linked to from the "About me" page, which is available for all control and intervention group participants who have logged in.

Title: An incident has occurred

Subtitle: This form can be used to report problems that you have experienced through taking part in the NEON Trial. We use this information to help us monitor the safety of NEON and of individual participants. Please tell us as much as you can, as this will help us in responding effectively. Any information you tell us will be logged, and reported to the NEON Chief Investigator and others.

Q1: What has happened? [Response options: I experienced something that threatened my life, I was admitted to hospital, I had to stay in hospital longer than planned or expected, I have been disabled or incapacitated, I have experienced something else I want to tell you about (opens text box labelled: Please describe what you experienced)]

Q2: In your opinion was this caused by your participation in a NEON trial? [Response options: yes, unsure, no]

[If the response to Question 2 is "yes" or "unsure"] Q3: When did this occur? Please give us your best guess if you cannot remember accurately. [Date entry field]

Q4: Where did this occur? [Multiline free text]

[If the response to Question 2 is "yes" or "unsure"] Q5: Please tell us how you think the trial might have caused this. [Multiline free text]

Q6: How is it best for us to contact you if we need more information? [Multiline free text]

Response on completion: Thank you for taking the time to complete this form. The information that you have provided has been immediately passed to the NEON Chief Investigator and study coordinator, and will be processed rapidly.

[Notes: Q1 and Q2 defaults to "Select an answer from the list", and a response option must be selected before the form can be submitted. Q3 defaults to a blank date, and a date must be entered before the form can be submitted. Q4-Q8 default to blank text if enabled, and some text must be entered before the form can be submitted.
